# Connectomic and Surface-Based Morphometric Correlates of Acute Mild Traumatic Brain Injury

**DOI:** 10.3389/fnhum.2016.00127

**Published:** 2016-03-29

**Authors:** Patrizia Dall'Acqua, Sönke Johannes, Ladislav Mica, Hans-Peter Simmen, Richard Glaab, Javier Fandino, Markus Schwendinger, Christoph Meier, Erika J. Ulbrich, Andreas Müller, Lutz Jäncke, Jürgen Hänggi

**Affiliations:** ^1^Bellikon Rehabilitation ClinicBellikon, Switzerland; ^2^Division Neuropsychology, Department of Psychology, University of ZurichZurich, Switzerland; ^3^Division of Trauma Surgery, University Hospital ZurichZurich, Switzerland; ^4^Department of Traumatology, Cantonal Hospital AarauAarau, Switzerland; ^5^Department of Neurosurgery, Cantonal Hospital AarauAarau, Switzerland; ^6^Interdisciplinary Emergency Centre, Baden Cantonal HospitalBaden, Switzerland; ^7^Department of Surgery, Waid Hospital ZurichZurich, Switzerland; ^8^Institute of Diagnostic and Interventional Radiology, University Hospital ZurichZurich, Switzerland; ^9^Brain and Trauma Foundation GrisonChur, Switzerland; ^10^International Normal Aging and Plasticity Imaging Center, University of ZurichZurich, Switzerland; ^11^Center for Integrative Human Physiology, University of ZurichZurich, Switzerland; ^12^University Research Priority Program, Dynamic of Healthy Aging, University of ZurichZurich, Switzerland

**Keywords:** mild traumatic brain injury, structural connectome, connectivity analysis, cortical surface area, multimodal MRI, subjective symptoms

## Abstract

Reduced integrity of white matter (WM) pathways and subtle anomalies in gray matter (GM) morphology have been hypothesized as mechanisms in mild traumatic brain injury (mTBI). However, findings on structural brain changes in early stages after mTBI are inconsistent and findings related to early symptoms severity are rare. Fifty-one patients were assessed with multimodal neuroimaging and clinical methods exclusively within 7 days following mTBI and compared to 53 controls. Whole-brain connectivity based on diffusion tensor imaging was subjected to network-based statistics, whereas cortical surface area, thickness, and volume based on T1-weighted MRI scans were investigated using surface-based morphometric analysis. Reduced connectivity strength within a subnetwork of 59 edges located predominantly in bilateral frontal lobes was significantly associated with higher levels of self-reported symptoms. In addition, cortical surface area decreases were associated with stronger complaints in five clusters located in bilateral frontal and postcentral cortices, and in the right inferior temporal region. Alterations in WM and GM were localized in similar brain regions and moderately-to-strongly related to each other. Furthermore, the reduction of cortical surface area in the frontal regions was correlated with poorer attentive-executive performance in the mTBI group. Finally, group differences were detected in both the WM and GM, especially when focusing on a subgroup of patients with greater complaints, indicating the importance of classifying mTBI patients according to severity of symptoms. This study provides evidence that mTBI affects not only the integrity of WM networks by means of axonal damage but also the morphology of the cortex during the initial post-injury period. These anomalies might be greater in the acute period than previously believed and the involvement of frontal brain regions was consistently pronounced in both findings. The dysconnected subnetwork suggests that mTBI can be conceptualized as a dysconnection syndrome. It remains unclear whether reduced WM integrity is the trigger for changes in cortical surface area or whether tissue deformations are the direct result of mechanical forces acting on the brain. The findings suggest that rapid identification of high-risk patients with the use of clinical scales should be assessed acutely as part of the mTBI protocol.

## Introduction

Research into mild traumatic brain injury (mTBI) has frequently highlighted difficulties in interpreting results derived from a unique biomarker or neuroimaging technique to explore brain injuries (Shenton et al., 2012; Ling et al., 2013; Dodd et al., 2014). The need for MRI modalities sensitive to both gray (GM) and white matter (WM) remains urgent, in particular to capture the subtle and dynamic neuropathology of the acute phase following mTBI. Disrupted WM integrity of cortico-cortical and subcortico-cortical pathways and the consequences of diffuse axonal injuries (DAI), a result of shear-strain deformations in the injured brain, have been hypothesized as the acute pathophysiological mechanisms underlying mTBI (Arfanakis et al., 2002; Inglese et al., 2005). Concurrently, especially in animal studies, subtle morphological anomalies in GM structures as synapse reduction and dendritic degeneration have been postulated as the pathogenic basis of acute mTBI (Gao and Chen, 2011; Shenton et al., 2012).

Progress in neuroimaging has provided promising instruments capable of identifying valid early biomarkers of mTBI and enhancing our understanding of neurological substrates behind physical, emotional, and cognitive complaints associated with mTBI. Such complaints include impairments in processing speed, memory, and executive functions that can last for weeks to months. Nowadays, two established and highly sensitive modalities, diffusion tensor imaging (DTI) and high resolution T1-weighted MRI are able to detect structural alterations in WM and GM, respectively. The combination of DTI-based fiber tractography and graph-theoretical network analysis has opened new powerful possibilities to explore the brain's structural connectome (Hagmann et al., 2008; Bullmore and Sporns, 2009; Hänggi et al., 2014). In this graph-based approach, brain complexity is mapped as a widely distributed network of nodes and edges, representing brain regions and its axonal tracts (Zalesky et al., 2010). In this framework, the strength of the anatomical link between two nodes is measured as the total number of interconnecting fibers. This novel approach has already offered insights into structural network changes after TBI, revealing disrupted network efficiency (Caeyenberghs et al., 2012, 2014; Kim et al., 2014). However, these results are based on moderate-to-severe TBI cases and limited to chronic brain conditions. Only one DTI-based network study has investigated structural connectivity changes early after mTBI. This study with children demonstrated evidence of correlation between symptom severity as assessed by the post-concussion symptom scale score, and nodal degree of two frontal hubs (Yuan et al., 2015). Numerous studies have disclosed WM changes at different intervals following mTBI by applying traditional DTI approaches, which examine common diffusion metrics such as fractional anisotropy and mean diffusivity. However, these studies are inconsistent in two aspects. First, findings on acute and semi-acute stages of mTBI are very heterogeneous in contrast to almost convergent observations of reduced fractional anisotropy in chronic stages (Shenton et al., 2012; Dodd et al., 2014; Ilvesmaki et al., 2014).

Second, the correlation between alterations in structural integrity and symptom severity following mTBI remains inconsistent with some studies providing evidence of an existing association in the acute (Bazarian et al., 2007; Wilde et al., 2008) or subacute post-injury phase (Messé et al., 2011, 2012; Smits et al., 2011) and others reporting no association (Lange et al., 2012; Ling et al., 2012, 2013; Wäljas et al., 2014, 2015). At the same time, little is known about the early morphometric changes occurring in human GM structures after mTBI. Recently, automated T1-weighted surface-based morphometric (SBM) analysis has allowed the distinct exploration of cortical surface area, thickness, and volume alterations over the entire cortex. The majority of morphometric studies have investigated mTBI patients in later phases (Chen et al., 2008; Tremblay et al., 2013; Zhou et al., 2013; Tate et al., 2014). Only a few studies have focused on cortical structures at the initial phase and significant differences in volume or thickness between trauma and control groups was not found (Toth et al., 2013; Narayana et al., 2015). To our knowledge, no studies to date have completed structural multimodal MRI evaluations on a large sample of mTBI patients to elucidate acute WM and GM alterations, their interrelationships, or their associations with clinical aspects and cognitive functioning.

The current study presents the first investigation of the structural connectome using DTI-based network analysis and explores distinct morphological features using high-resolution T1-weighted MRI in mTBI adults within 7 days post-injury.

We expected to find alterations in structural connectivity and cortical morphology in patients compared to matched healthy controls (HC) and also predicted correlations between WM/GM changes and the severity of subjective complaints. In addition, we hypothesized relationships between these two trauma-induced structural alterations. Finally, we predicted that WM and GM alterations would be associated with cognitive performance.

## Materials and methods

### Subjects

A total of 118 subjects participated in this cross-sectional study between February 2012 and March 2014. We initially included 60 patients with recent mTBI and 58 HC. The HC were matched to the patients with respect to sex, age, years of education, and handedness. Patients were recruited in the emergency departments of four hospitals in the German region of Switzerland, and were selected according to the European Federation of Neurological Societies guidelines (Vos et al., 2012). Inclusion criteria comprised a Glasgow coma scale (GCS) score of 13–15 at hospital admission, a normal posttraumatic CT, and at least one of the following characteristics: (1) loss of consciousness < 30 min; (2) presence of a qualitative alteration in mental status, such as confusion, disorientation or dizziness at the time of incident; (3) post-traumatic amnesia < 60 min; and (4) retrograde amnesia < 30 min. Additionally, participants were excluded from both groups if they presented any of the following exclusion criteria: history of neurologic or psychiatric disease, history of neurosurgery, previous TBI, present or past drug or alcohol abuse, contraindications to MRI, or ages under 18 or over 64 years. Nine mTBI patients and five HC were subsequently excluded from the study because of incidental brain anomalies (two patients and one HC), excessive MRI-related motion artifacts (two patients), previous history of neurologic or psychiatric disturbance (two patients and three HC), and questionable diagnosis of mTBI (one patient). Two patients and one HC were regarded as non-compliance because they could not guarantee their participation for the assessment after 1 year (since the present work is part of a longitudinal study). Consequently, data of 51 patients and 53 HC were subjected to further analyses. All patients were imaged and clinically assessed within 7 days post-injury. The HC completed the same neuropsychological and MRI assessment as the patients, except for the conventional CT in the emergency department and the general neurological examination by licensed neurologists. The Swiss cantonal research ethics committees approved the study protocol and written informed consent was obtained from all participants prior to study enrolment. All subjects were equally reimbursed to make up for income lost due to study participation.

### Neuropsychological assessment

The compilation of clinical questionnaires and validated cognitive tests is summarized in Supplementary Table 1. Early post-concussive symptoms were assessed using the well-established Rivermead Post Concussion Symptoms Questionnaire (RPQ) (King et al., 1995). A German in-house translation of the RPQ was produced. This symptom checklist was selected as a core measure based on its capacity to detect somatic, cognitive, and emotional changes in patients with mTBI. The RPQ investigates the severity of 16 commonly reported post-traumatic symptoms, grading each item from 0 (not experienced) to 4 (severe problem). Patients rated perceived changes in performance occurred over the past 24 h, and HC were asked to rate the current severity of each symptom in comparison with their usual levels of functioning. The total RPQ score was calculated by summing all 16 items with a score of 2 or greater, as a score of 1 indicates that a symptom is not more problematic than before the trauma. In addition, two scales were used to assess emotional symptomatology: the German version (Hautzinger et al., 2006) of the Beck Depression Inventory 2nd edition BDI-II (Beck et al., 1996) was selected to control for manifestations of depression; and the German version (Margraf and Ehlers, 2007) of the Beck Anxiety Inventory BAI (Beck and Steer, 1993) was chosen to evaluate anxiety symptoms in response to mTBI. Further details regarding the neuropsychological test battery applied in the present study are provided in the Supplementary Materials online.

### Magnetic resonance imaging data acquisition, preprocessing, and construction of the network

Data acquisition, preprocessing of DTI and SBM data and the construction of the structural networks are described in detail in the Supplementary Materials. Briefly, a 3 T scanner was used to acquire DTI, T1-weighted, and conventional MRI scans.

Presence of intracranial injury was determined by the evaluation of conventional MR scans (T1, T2, FLAIR, and SWI) and examined by the same radiologist (E.J.U.) not blinded to mTBI-diagnosis. In cases where any salient features were noted, the subjects were excluded from the sample.

Preprocessing of the diffusion-weighted MRI data is described in the Supplementary Materials and was performed with FSL tools (FMRIB software library; version 5.0.6; http://www.fmrib.ox.ac.uk/fsl/) (Smith et al., 2004) such as the FDT (FMRIB diffusion toolbox; version 3.0) (Behrens et al., 2003). For deterministic fiber tractography, we used the Diffusion Toolkit (DTK, version 0.6.2.1) and TrackVis software (version 0.5.2.1; http://trackvis.org/) (Park et al., 2009).

Cortical surface reconstruction is described in the Supplementary Materials and was performed with the FreeSurfer image analysis suite (version 5.3.0), which is documented and freely available online (http://surfer.nmr.mgh.harvard.edu/). The technical details of these procedures are described in prior publications (Dale et al., 1999; Fischl et al., 1999a,b, 2001, 2002, 2004a,b; Fischl and Dale, 2000). Reconstructed surface models were visually checked for accuracy by an experienced imaging analyst (J.H.) and no manual interventions were necessary.

### Statistical analyses

#### Correlations with symptom severity and group comparisons

Network-based statistical analysis of the DTI data was performed using the network-based statistic (NBS) tool (https://sites.google.com/site/bctnet/comparison/nbs). NBS is a method for controlling the family-wise error rate when mass univariate testing is performed at every connection comprising the graph or network (Zalesky et al., 2010). Whole-brain analyses were performed with the *component extent* option in NBS, which is suitable for detecting an experimental effect that is relatively weak but that extends to encompass many connections (Zalesky et al., 2010).

Furthermore, based on T1-weighted MRI data, cortical surface area, thickness and volume across both hemispheres were investigated using the general linear model implemented in FreeSurfer's MRI_GLMFIT tool (http://surfer.nmr.mgh.harvard.edu/fswiki). Total RPQ score was correlated with both the number of reconstructed streamlines at each connection between the 90 nodes of the network and with cortical surface area, thickness and volume. Correlations were performed within each group separately and assessed with Pearson's correlation using IBM SPSS statistics software (version 22.0, http://www-01.ibm.com/software/analytics/spss/). Moreover, the above-mentioned WM and GM values were whole-brain compared between mTBI patients and HC. The data for patients and HC were in the same reference space and thus no transformation between spaces was needed when the correlation was applied over both groups. Finally, the number of streamlines of significant connections resulting from the DTI-based network analysis and the mean values within the resulting clusters derived from the SBM analysis were extracted. These data were then exported to SPSS Statistics, intercorrelated, and also correlated with cognitive test performance. The significance level of 0.05 was corrected for multiple comparisons by applying 5000 permutations of the RPQ value across subjects for the correlations and subjects across groups for the group comparisons as implemented in NBS and FreeSurfer. We included age as a covariate of no interest for the correlation analyses, as age was regarded as potential confounder due to its documented effect on WM and GM. Since the patient and HC groups were rigorously matched for sex, age, education, and handedness, it was not necessary to correct for these confounders in the group analysis. Group comparisons were performed with both the whole sample (51 patients and 53 HC) and with a reduced subsample comprising 30 patients and 30 HC. The reduced patients group, which showed moderate-to-severe symptoms (RPQ score ≥ 10), was selected assuming that patients with little or no self-reported symptomatology in the acute stage post-injury might show minimal or no changes in structural connectivity and surface-based morphometric measures of the brain. The 30 HC were rigorously selected and matched in sex, age, and education with the 30 patients.

Demographic measures, global brain indices and performance on neuropsychological assessment between groups were evaluated using *t*-tests for independent samples and Chi square tests. Based on studies reporting poorer cognitive performance in the initial phase after mTBI compared to controls (Rosenbaum and Lipton, 2012), differences between groups in neuropsychological assessment were analyzed using one-tailed *t*-tests. Group comparisons of 20 clinical and cognitive measures were subjected to Bonferroni correction taking into account the mean correlation (http://www.quantitativeskills.com/sisa/calculations/bonfer.htm) of the 20 variables tested.

## Results

### Demographics, clinical characteristics, and global brain measures

Demographic characteristics, global brain measures and the most relevant clinical variables are presented in Table 1.

**Table 1:** **Characteristics of patient and control groups**.

	**Patients (*****n*** = **51)**			**Controls (*****n*** = **53)**			***p*-value (two-sided)**
**Gender (female/male)**	**19/32**			**20/33**			**0.960**
**Handedness (right/left)**	**46/5**			**44/9**			**0.284**
	**Mean**	**SD**	**Range (Min./Max.)**	**Mean**	**SD**	**Range (Min./Max.)**	
Age (years)	34.5	12.4	18/61	34.2	12.1	18/60	0.887
Education (years)	12.5	2.5	8/18	12.8	2.4	7/19	0.509
GCS^a^	14.8	0.4	13/15	–	–	–	–
Number of mTBI in the past	0.6	0.9	0/4	0.4	0.8	0/3	0.271
**SURFACE-BASED MEASURES**							
Total surface area (cm^2^)	171.74	17.31	130.68/210.99	173.53	13.32	147.80/211.03	0.555
Mean cortical thickness (mm^2^)	2.47	0.11	2.21/2.79	2.45	0.11	2.19/2.66	0.300
Total gray matter volume (cm^3^)	639.89	65.32	479.70/767.50	639.93	53.58	533.99/735.49	0.998
Total white matter volume (cm^3^)	483.51	59.40	364.66/592.62	487.01	38.37	398.69/557.22	0.723
Total subcortical gray matter volume (cm^3^)	61.58	5.33	48.50/75.89	61.20	4.12	53.62/72.60	0.687
**CONNECTIVITY MEASURES (90 NODES)**							
Total number of streamlines	2,156,105	244,298	1,501,810/2,661,329	2,145,401	171,886	1,751,206/2,519,561	0.797
Streamlines omitted	1,372,326	135,137	1,106,589/1,628,276	1,355,014	117,186	1,130,381/1,573,362	0.486
Streamlines used to populate matrix	832,291	145,735	445,646/1,142,486	836,944	95,077	609,833/1,110,992	0.848
Selfloops	466,828	70,021	290,453/613,120	463,774	43,599	386,019/574,392	0.791
**NEUROPSYCHOLOGICAL ASSESSMENT**							
RPQ (total score)^b^	13.98	10.80	0/46	2.85	4.15	0/20	<0.001^c^
Go/Nogo (ms)	398.71	67.92	246/558	370.38	44.54	286/497	0.007^c^
Divided attention, visual (ms)	803.12	104	628/1075	754	87.34	605/985	0.005^c^

a GCS, Glasgow coma scale;

b RPQ, Rivermead Post-Concussion Symptoms Questionnaire;

c one-sided differences between groups after adjustment for multiple testing. The Bonferroni-corrected significance level was set at p < 0.0047 taking into account the mean correlation (r = 0.21) of the 20 variables tested. All other neuropsychological scores are illustrated in Supplementary Table 1.

Of the 51 patients, 32 were male, and the mean age at injury was 34.5 years (SD = 12.4 years, range 18–61 years). Glasgow coma scale (GCS) score at admission in the emergency department was 15 in 42 patients, 14 in eight patients and 13 in one patient.

**Figure 1:**
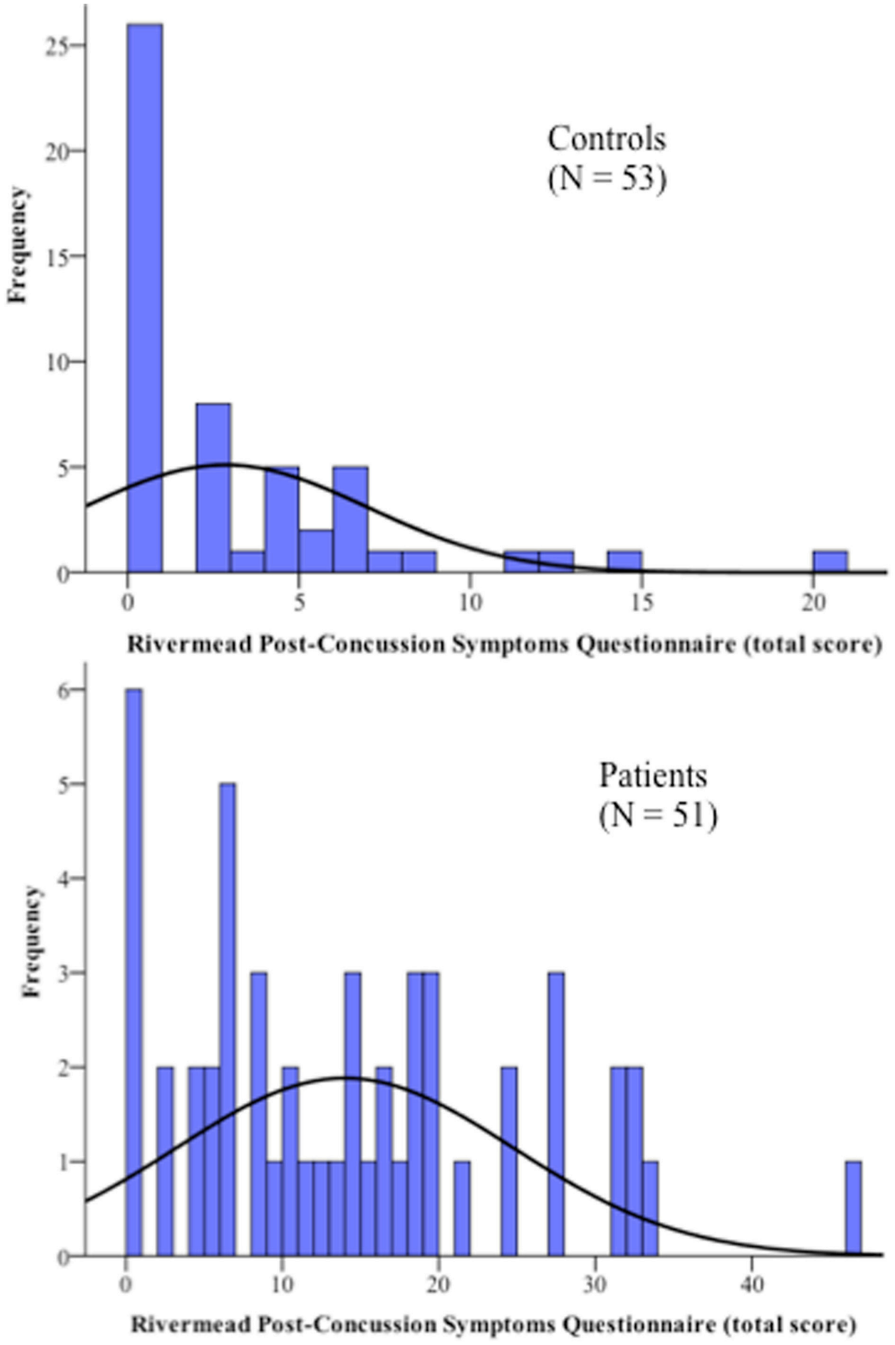
**Distribution of the complaints of all participants as measured by the Rivermead Post-Concussion Symptoms Questionnaire**.

The subsample of 30 mTBI subjects with more severe symptoms did not differ with respect to GCS from the remaining 21 patients reporting fewer or less severe symptoms. No significant differences were observed between patients and HC regarding key demographic variables or past exposure to mTBI. On average, the patients underwent MRI scans at 4.9 days (SD = 1.47 days) and were clinically assessed at 5.3 days (SD = 1.62 days) after injury. Clinically, the only between-group difference that reached significance was the RPQ score. Total RPQ score was significantly higher in the mTBI group (mean = 13.9, SD = 10.8) than in HC (mean = 2.8, SD = 4.1) at 1-week post-injury (Figure 1).

However, there is an overlap in the lower severities of the complaints between patients and HC, where 37% of the patients reported few or no symptoms, comparable to the response pattern of the control group. This result is in line with other mTBI studies (Sterr et al., 2006; Laborey et al., 2014). The large distribution of total RPQ score within the mTBI group supported our decision to also perform group comparisons with a reduced subsample of 30 patients suffering from moderate-to-severe symptoms (RPQ ≥ 10) and the 30 matched HC.

For neuropsychological measures, we found a statistical trend in two tasks, namely the Go/Nogo and the divided attention task, suggesting reduced performance in patients compared with HC (Table 1). Finally, no significant differences between groups were found for total GM or WM volume, total subcortical GM volume, total cortical surface area, or mean cortical thickness.

### Structural connectome

**Figure 2:**
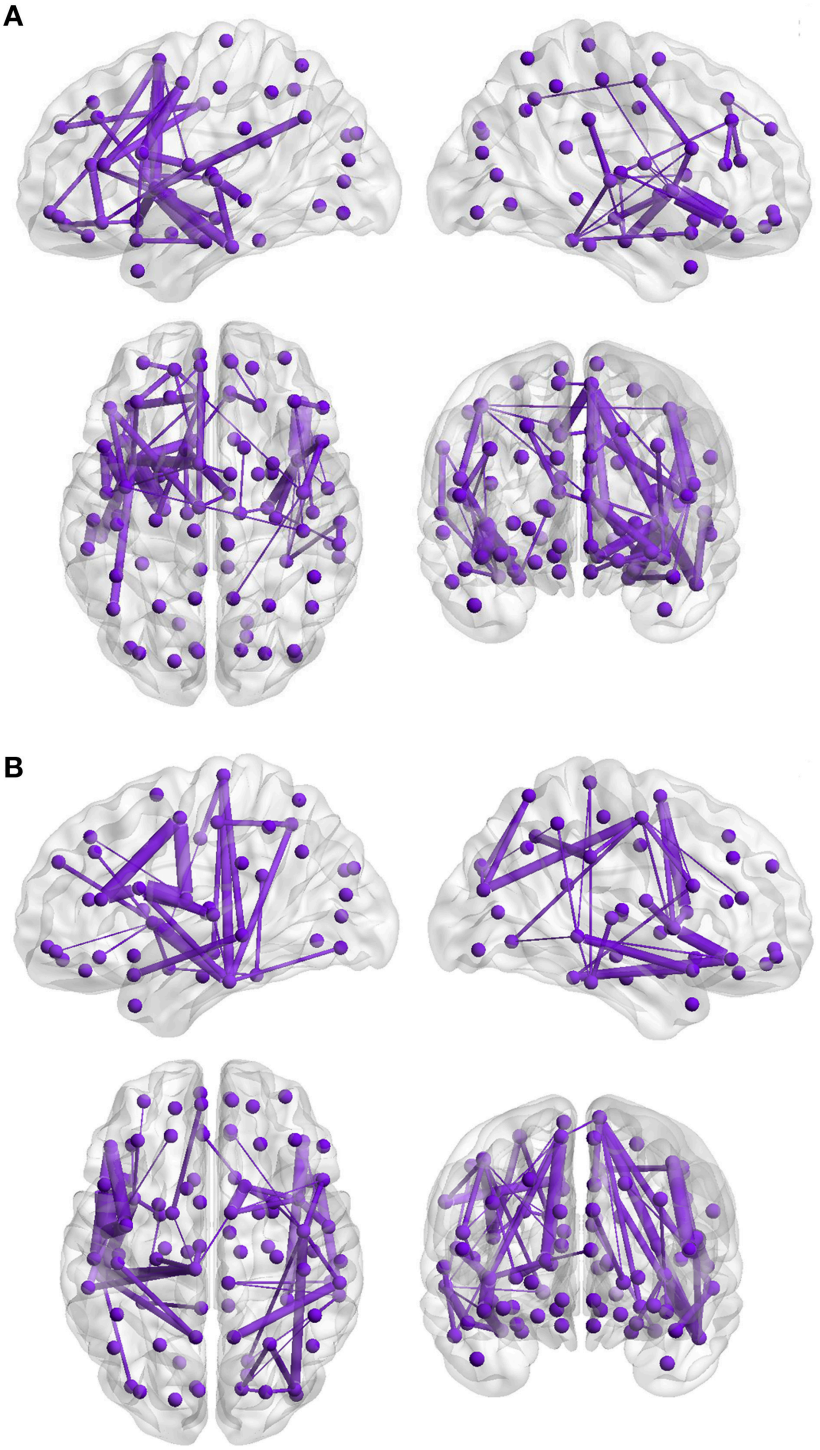
**Inverse correlation between structural connectivity and Rivermead Post-Concussion Symptoms Questionnaire scores of the patients and group comparison**. The 3D visualization of the network (Xia et al., 2013) shows a lateral section of the left and the right hemispheres (upper panels), horizontal and coronal sections (lower left: superior perspective, lower right: anterior perspective). The violet-colored dots correspond to the 90 cortical and sub-cortical automated anatomical labeling regions. The violet lines represent the supra-threshold connections of the subnetwork. The thickness of the lines represents the absolute connectivity strength of each connection. **(A)** The connectivity strength of one subnetwork is inversely correlated with the total RPQ score of the patients (*n* = 51, mean *r* = −0.31, *p* = 0.045 corrected). **(B)** Reduced structural connectivity in the subgroup of 30 mTBI patients compared to 30 matched controls (Cohen's *d* = 0.95, *p* = 0.027).

First, the structural connectivity of a single subnetwork was inversely related to the total RPQ score of the patients (mean *r* = −0.31, *p* = 0.045 corrected, primary threshold *t* = 1.80). The extent of this altered subnetwork comprised 59 inter-regional connections distributed over 50 nodes (Figure 2A, Supplementary video 1). The altered set of connections encompassed bilateral frontal, parietal, temporal, and subcortical regions such as hippocampus, thalamus and caudate nucleus. Connections going to or from the frontal cortex showed the highest concentration, i.e., 31 out of 59 connections (Supplementary Table 2). Further exploration of the dysconnected subnetwork revealed that the most affected connections were intra-hemispheric, with only 6 connections being inter-hemispheric. The nodes with the most connections related to severity of post-injury symptoms were located in the left insula (8 connections), left anterior cingulate (6 connections), left supplementary motor area (6 connections), and right insula (7 connections). Next, the same correlation between total RPQ and structural connectivity but based only on the 30 more symptomatic subjects was performed. Similar as for the entire patient group, the association was negative, but did not reach statistical significance (*p* = 0.180).

Finally, no correlation between connectivity strength and total RPQ score was observed in the HC group (*n* = 53), although some HC also indicated post-concussion-like complaints (Figure 1).

Second, the results of the group comparison revealed no significant differences when considering the entire sample (*n* = 51 patients/53 HC, *p* = 0.626), whereas investigation of the more severe subgroup revealed a single dysconnected subnetwork with reduced connectivity strength in the 30 patients compared to their 30 HC (Cohen's *d* = 0.95, *p* = 0.027, Figure 2B, Supplementary Table 3). The dysconnected subnetwork was composed of 57 connections distributed over 48 nodes; the primary threshold was set to *t* = 1.81. A closer examination of the subnetwork pattern revealed the presence of 11 connections identical to those resulting in the correlation analysis. No subnetwork showed greater structural connectivity in patients than HC.

### Surface-based morphometry

**Figure 3:**
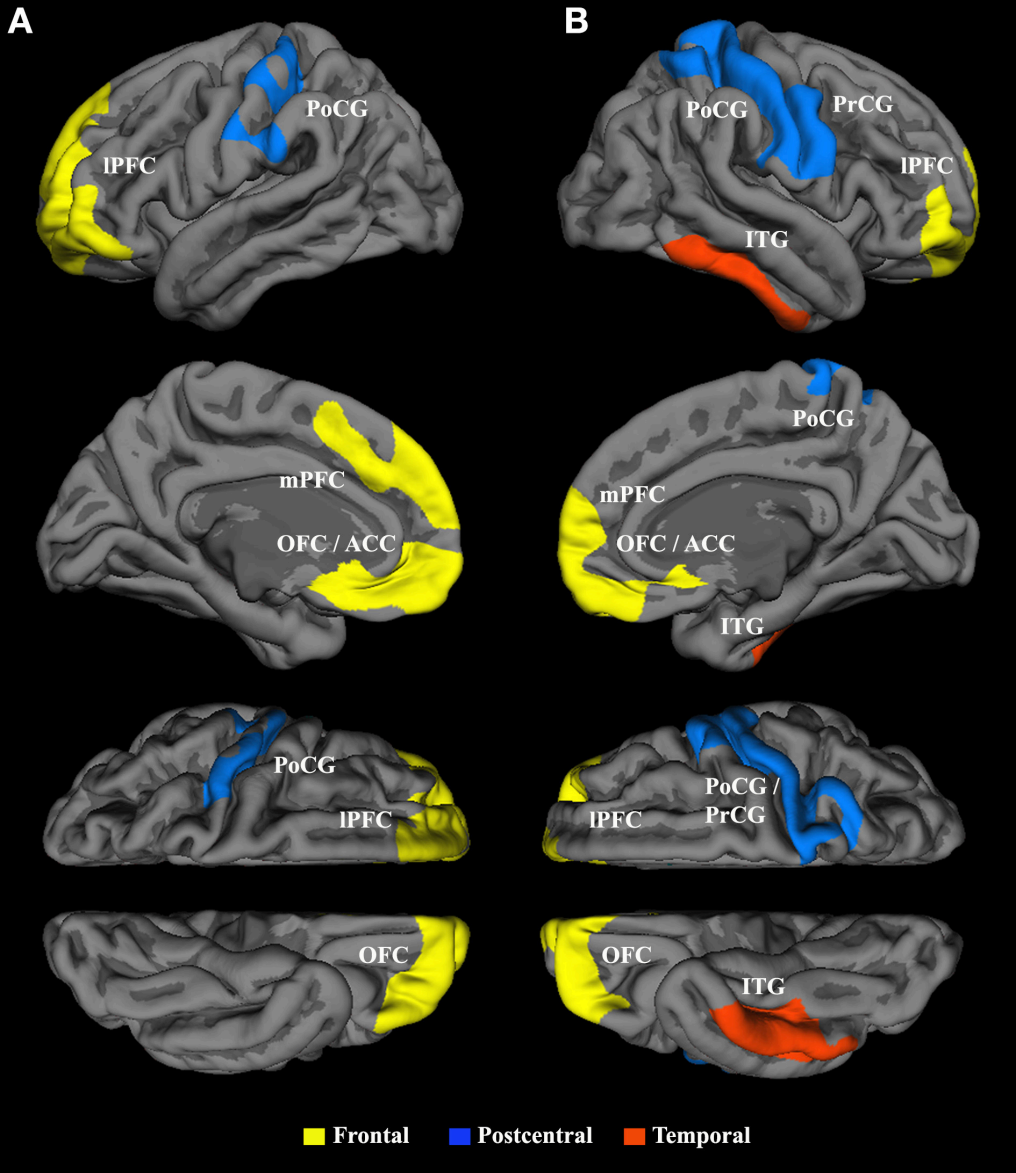
**Inverse correlation between cortical surface area and Rivermead Post-Concussion Symptoms Questionnaire scores in the patient group (*n* = 51)**. Panels of the top row show the lateral hemispheres (**A** = left, **B** = right), whereas the second row shows the medial hemispheres. The third and bottom rows represent the superior and inferior views, respectively. Bilateral frontal clusters (yellow) encompass the lateral and medial prefrontal cortex, the orbitofrontal cortex and the anterior cingulate cortex. The parietal cluster (blue) in the left hemisphere includes the postcentral gyrus (PoCG) together with parts of the central sulcus, while the frontoparietal cluster (blue) in the right hemisphere stretched over the PoCG and the precentral gyrus. The red cluster in the right hemisphere comprises the inferior temporal gyrus. Only clusters exceeding a cluster-wise corrected probability of *p* < 0.05 are shown.

First, correlation analysis conducted within the mTBI group revealed an inverse association between total RPQ score and cortical surface area in five clusters. Bilateral clusters in the frontal and parietal cortices and one cluster in the right inferior temporal gyrus (ITG) were found to be negatively correlated with early mTBI-related symptoms, showing that patients reporting more severe symptoms had lower cortical surface area (Figure 3, Table 2, Supplementary Video 2A for the left and Supplementary Video 2B for the right hemisphere). In the left hemisphere, a corresponding temporal cluster with reduced surface area was also detected, which did not, however, reach statistical significance after correction for multiple comparisons (*r* = −0.11, *df* = 48, cluster-wise corrected *p*-value, CWP = 0.22). The largest cluster was identified in the frontal region of the left hemisphere. The clusters with the strongest associations between RPQ and area were located bilaterally in the frontal cortex and in the right frontoparietal cortex. The RPQ score did not have any association with cortical thickness, but correlation with cortical volume revealed the same clusters as in the analysis of cortical surface area (with the exception of one cluster in the right precuneus instead of the right frontal cortex). No significant correlation between RPQ score and any of the surface-based measures was found in the HC. Driven by the NBS finding, post hoc ROI analyses focusing on the insula were performed, revealing negative correlations with symptoms level in both cortical surface areas (left, *r* = −0.270, *p* = 0.029; right, *r* = −0.212, *p* = 0.070; uncorrected). Similar correlations were found with cortical volume.

**Table 2:** **Inverse correlation between surface area and total Rivermead Post-Concussion Symptoms Questionnaire score in the patients (*n* = 51)**.

**Measure and anatomical location**	**Cluster name**	**Cluster color in Figure 3**	**Cluster size (mm^2^)**	**Number of vertices**	**MNI coordinates**			***t*-value (df = 48)**	**CWP**	**Correlation coefficient (*R*^2^)**
					**x**	**y**	**z**			
**CORTICAL SURFACE AREA (LEFT)**										
Lateral prefrontal cortex (lPFC) Medial prefrontal cortex (mPFC) Orbitofrontal cortex (OFC) Anterior cingulate cortex (ACC)	Left frontal	Yellow	7313.6	9921	−19.4	62.9	−12.9	3.81	0.0002	−0.48 (0.23)
Postcentral gyrus (PoCG)	Left parietal	Blue	2280.1	5055	−34	−35.1	62.7	2.18	0.017	−0.3 (0.09)
**CORTICAL SURFACE AREA (RIGHT)**										
Postcentral gyrus (PoCG) Precentral gyrus (PrCG)	Right frontoparietal	Blue	5728.9	12586	54.1	−2	44.1	3.81	0.0002	−0.48 (0.23)
Lateral prefrontal cortex (lPFC) Medial prefrontal cortex (mPFC) Orbitofrontal cortex (OFC) Anterior cingulate cortex (ACC)	Right frontal	Yellow	3580.4	4608	5.3	50.8	−21.4	3.44	0.0006	−0.45 (0.20)
Inferior temporal gyrus (ITG)	Right temporal	Red	2449.1	3328	46.5	−31.6	−20.4	2.45	0.009	−0.33 (0.11)

MNI coordinates, coordinates of the maximum value found in the cluster within the MNI space; CWP, Clusterwise corrected p-value. [Only clusters exceeding a clusterwise corrected probability CWP of p < 0.05 are described].

**Figure 4:**
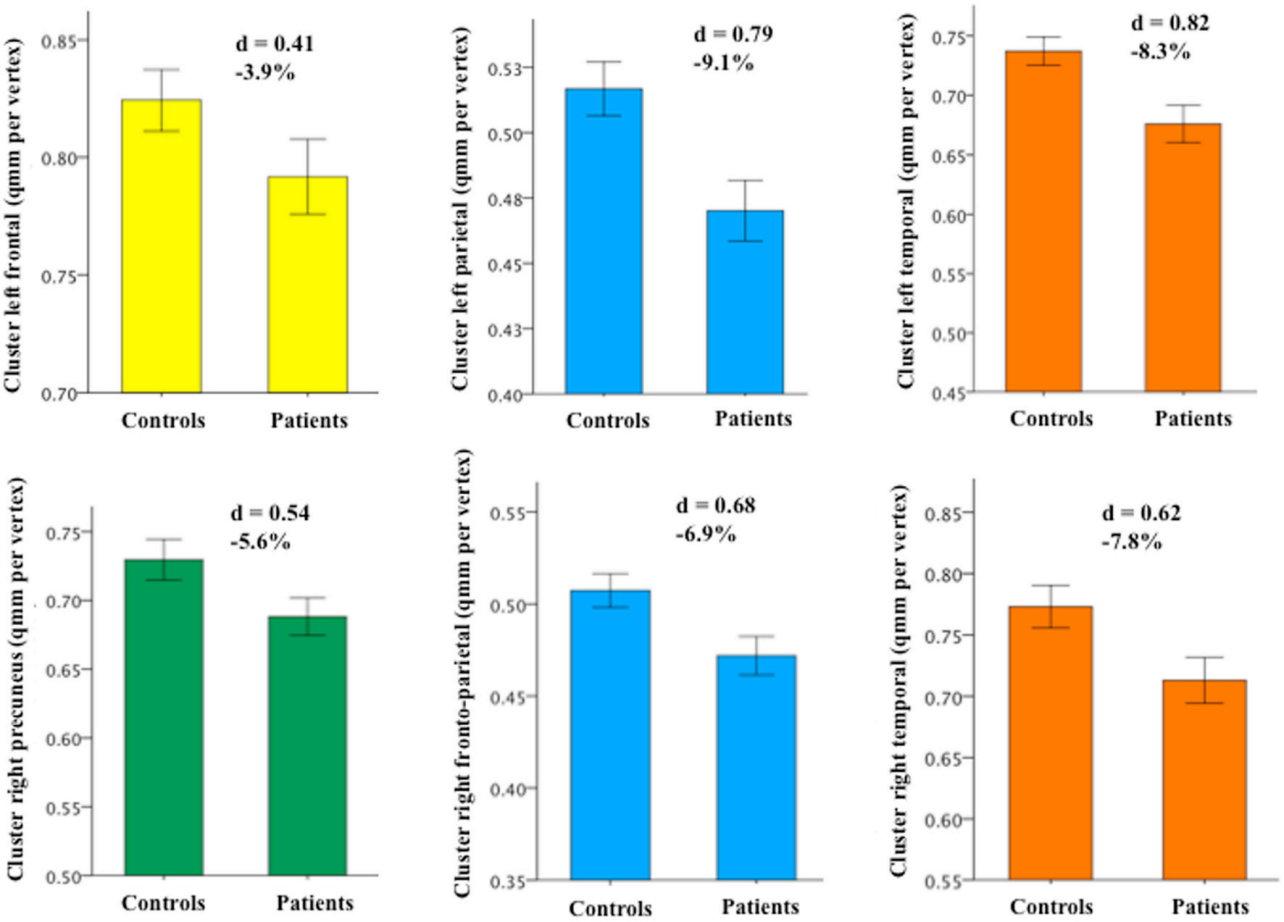
**Group differences in cortical surface area between the subgroup of 30 patients suffering from more severe symptoms and the 30 matched HC**. The same colors used in Figure 3 were assigned to the cortical clusters (the right precuneus is newly shown in green). Depending on the cluster, the percentage of surface area reduction in the patient group varied between 3.9 and 9.1%. [Error bar = ± standard error of mean SEM; the strength of the group difference (i.e., effect size) was assessed using Cohen's d score].

Next, cross-sectional whole-brain comparison of area, thickness and volume revealed no significant difference between traumatic group and HC (*p* > 0.15). Post-hoc we then compared the mean values between the groups within ROIs, defined as the clusters resulting from the correlation with the RPQ across both groups. To limit the number of comparisons, group analyses focused on the six surface area clusters that exhibited significant negative associations with total RPQ score across both groups (Supplementary Figure 1, Supplementary Table 4). When considering the entire sample, significant group differences were identified in the left parietal (*p* = 0.009) and left temporal cluster (*p* = 0.014; Supplementary Figure 2). The 30 patients suffering from more severe symptoms showed significant reductions in surface area in five of the six clusters compared to the 30 HC (Figure 4).

### Convergence of the white and gray matter findings

**Figure 5:**
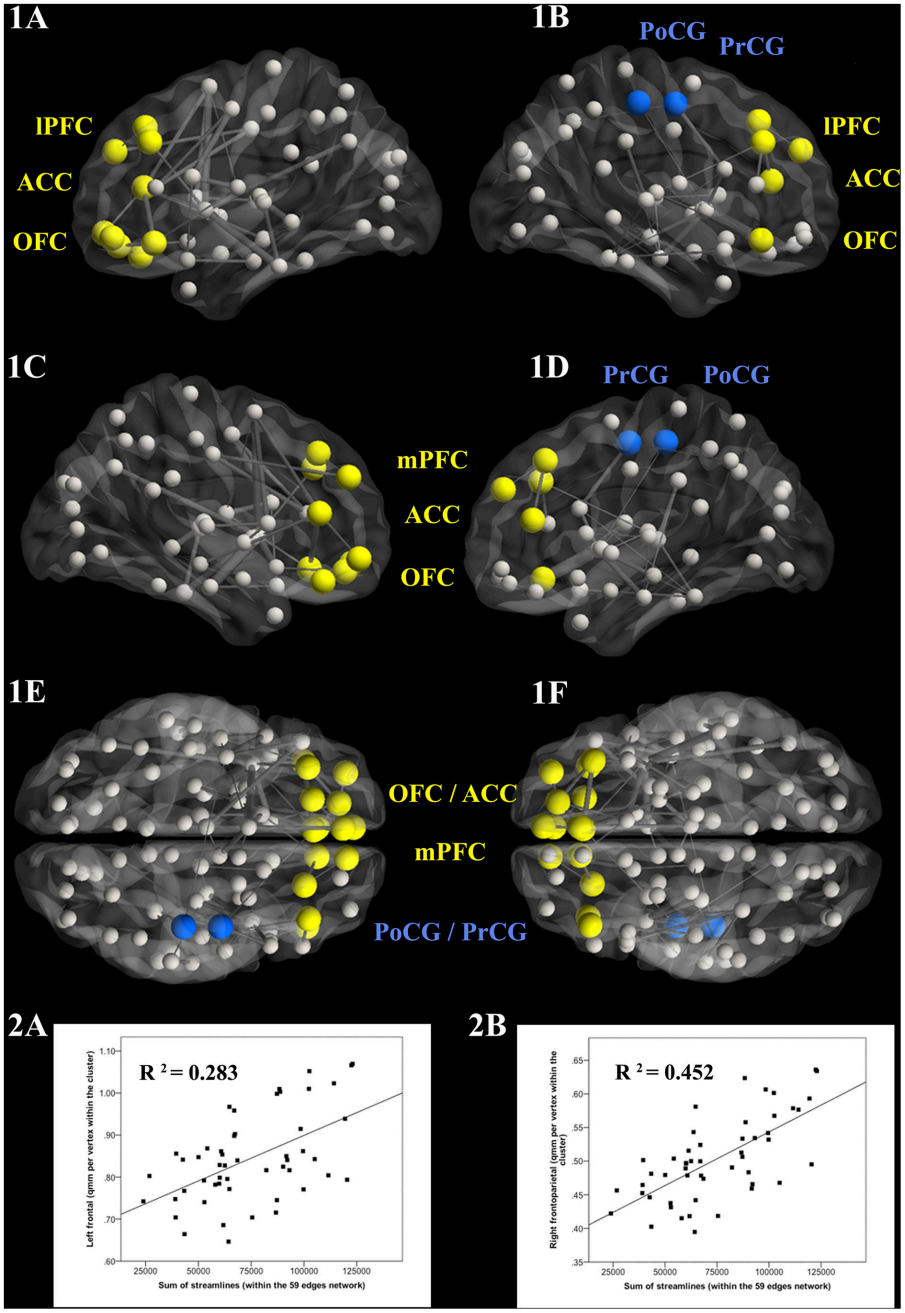
**Anatomical overlap of white and gray matter changes in the patients**. The 3D visualization of the network (Xia et al., 2013) includes the lateral view (**1A** = left, **1B** = right), the medial view **(1C,1D)**, and the horizontal superior **(1E)** and inferior **(1F)** views. The nodes in yellow and blue represent the brain regions of patients in whom changes were observed in both the surface- and connectivity-based analyses. The scatterplots illustrate the best correlations between surface area values and the sum of the streamlines within the 59-edge network **(2A,B)**.

Anatomically consistent structural changes were observed in bilateral frontal brain regions, i.e., in lateral (lPFC) and medial prefrontal cortices (mPFC), orbitofrontal cortex (OFC), and anterior cingulate cortex (ACC) as well as in the pre- (PrCG) and postcentral gyrus (PoCG) of the right hemisphere (Figures 5,1A–F). In the mTBI group, positive correlations (*p* < 0.001) between total number of reconstructed streamlines within the dysconnected subnetwork (59 edges) and surface area of the five clusters (mean surface area per vertex in mm^2^) have been found in the left frontal (*r* = 0.532), left parietal (*r* = 0.519), right frontal (*r* = 0.448), right frontoparietal (*r* = 0.673), and in the right temporal cluster (*r* = 0.439). Area reduction in the right frontoparietal and left frontal cluster explained up to 45 and 28%, respectively, of the variance in connectivity strength of the dysconnected subnetwork (Figures 5,2A,B).

### Correlations between structural brain alterations and cognitive performance

**Figure 6:**
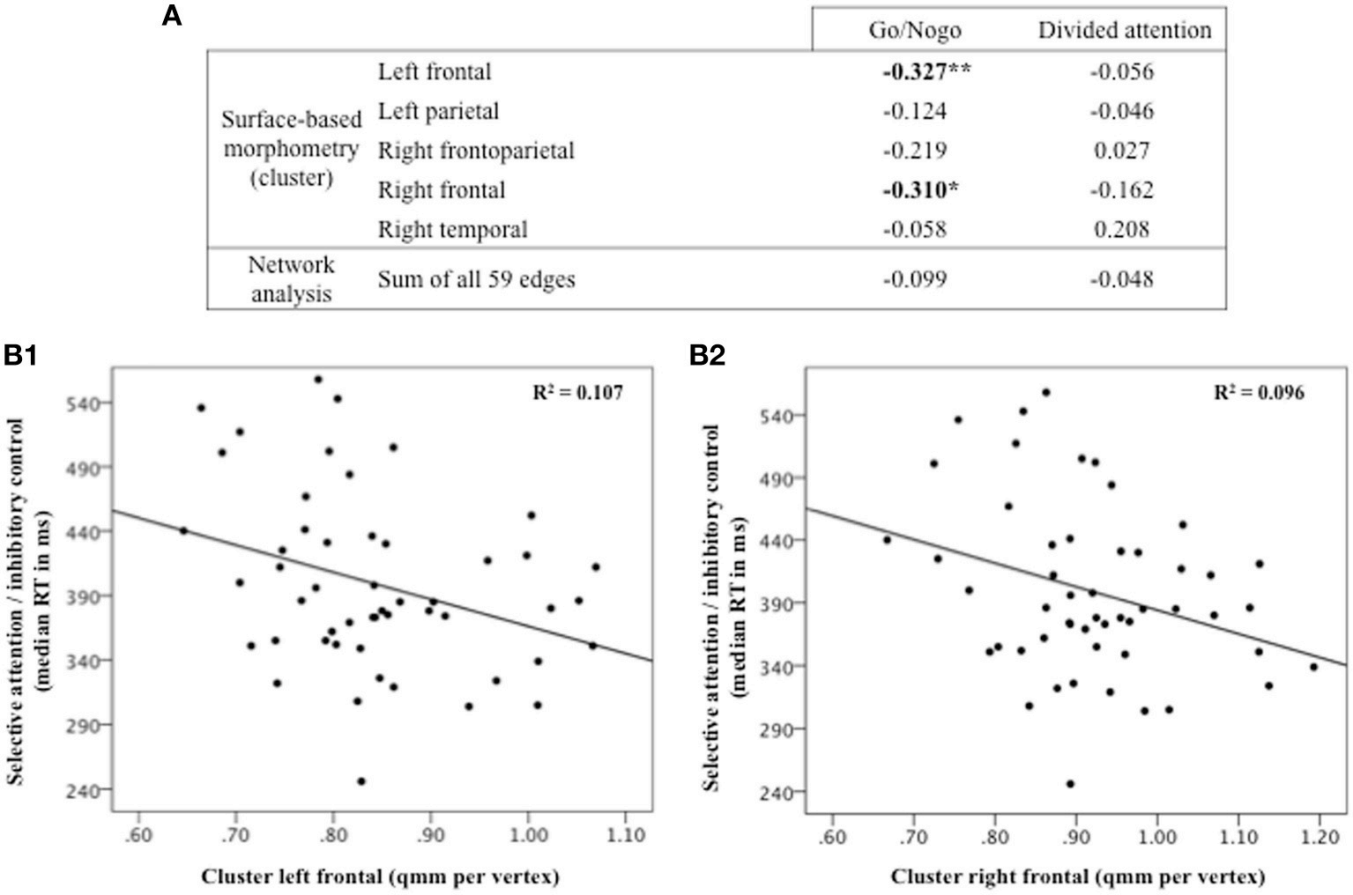
**Correlations between patients' performance on attentive/executive tasks and structural brain changes resulting from the relationship with total Rivermead Post-Concussion Symptoms Questionnaire score**. Significant effects of *p* < 0.05 are denoted by single asterisks and effects of *p* < 0.001 by double asterisks **(A)**. Poorer performance on the Go/Nogo task (longer reaction time) correlates with reduced surface area in the left **(B1)** and right **(B2)** frontal clusters.

Finally, correlations were performed to further explore whether cognitive performance in the mTBI group was potentially related to the dysconnected subnetwork or area reduction in the five clusters. Post hoc correlation analyses revealed that poorer performance on the Go/Nogo task (i.e., longer reaction times) was linked to reduced surface area in both the left (*r* = −0.327, *p* = 0.010) and right (*r* = −0.310, *p* = 0.013) frontal clusters (Figure 6). No other associations were observed.

## Discussion

This multimodal study is the first that investigates microstructural alterations in the adult brain within 7 days after mTBI by combining structural connectome analysis using DTI-based fiber tractography and SBM procedures using high-resolution T1-weighted MRI scans. We demonstrated that (i) both structural connectivity strength and cortical surface area show a similar potential to serve as a highly sensitive marker for the quantification of brain damage associated with subjective symptoms in acute mTBI patients; (ii) alterations in WM and GM are localized in similar brain regions and related to each other; and (iii) the reduction of area seen in frontal regions is correlated with poorer attentive-executive performance in the mTBI group.

### Network-based connectivity

Decreased structural connectivity within one subnetwork was significantly associated with an increase in self-reported complaints in the mTBI group. Descriptively, most of these edges (53 of 59) were intrahemispheric and involved both short and long connections. Although the dysconnected fibers appeared widespread when inspected by the naked eye, the involvement of frontal regions was highly pronounced (31 of 59 altered connections possessed a node in the frontal cortex). The insula also seems to play an essential role in the altered circuit, since the strongest connections related to self-reported complaints were identified in the insula. The close links of the frontal cortex and insula to symptom severity were found bilaterally and will be discussed later.

To date, only one graph-theoretical study using DTI has investigated connectivity in mTBI, although in children (Yuan et al., 2015). In nice agreement with our findings, the study demonstrated a correlation between symptom severity and nodal degree of the superior and middle frontal gyrus in the mTBI group.

Subsequently, we performed whole-brain comparison over the entire sample that yielded no significant group differences. Conversely, a reduction of structural connectivity within a single subnetwork comprising by 57 connections was detected in the more severe patient subgroup compared to their matched HC. The focus on this subgroup may have enhanced the detection of decreased WM integrity, an effect that was attenuated when the 21 less impaired patients were included. The importance of classifying patients according to symptom severity has already been put forth in classical DTI studies of mTBI patients (Messé et al., 2011; Wäljas et al., 2014), and other studies have even indicated that the level of symptom severity is more critical than the experience of mTBI *per se* (Sterr et al., 2006; Ponsford et al., 2012). As reported above, only one study on children with acute mTBI has applied graph theoretical analysis to investigate structural connectivity. They also observed a significant reduction in network efficiency compared to controls. Groupwise differences using tract-based spatial statics (TBSS) were previous largely reported in multiple, widespread white matter regions where the classical diffusion metrics as FA and mean diffusivity were significantly different in acute-subacute mTBI patients compared to controls (Messé et al., 2011, 2012; Toth et al., 2013; Croall et al., 2014; Zhu et al., 2014). Studies combining DTI tractography with graph theory to investigate patients with moderate-to-severe TBI have reported altered network organization and lower efficiency in the chronic phase after injury (Caeyenberghs et al., 2012, 2014; Kim et al., 2014). Caeyenberghs and colleagues suggested the idea of TBI as a dysconnection syndrome from a network perspective. Moreover, since recently TBI is considered an example of damage in which large-scale network alterations produce clinical impairments (Sharp et al., 2014). The present findings may support the extension of this assumption to the mild end of TBI. To our knowledge, this is the first study that demonstrates direct evidence for a dysconnection syndrome in adults acutely following mTBI.

As frequently reported in the literature over the last years (Shenton et al., 2012), biomechanically induced DAI could play a major role in mTBI. Our results are compatible with DAI and indicate that axonal damage may be responsible for the reduced connectivity strength in large-scale structural subnetworks.

### Surface-based morphometry

The surface-based morphometry analyses revealed that severity of complaints was inversely correlated with cortical surface area in mTBI patients. Area reductions associated with greater complaints were identified in five clusters located in left and right frontal cortices, left PoCG and right PoCG/PrCG, as well as in the right ITG. Similar results were detected for cortical volume, although we did not detect cortical thinning associated with clinical symptomatology. This implies that volume reduction was mainly attributable to area reduction, since volume is defined as the product of area and thickness.

Other mTBI studies have demonstrated a negative association between post-concussive symptoms and cortical morphology (Zhou et al., 2013; Albaugh et al., 2015). Anatomically strongly in line with our findings, these studies focused on the subacute-to-chronic phase following injury and only reported changes in volume or thickness. For example, a study of young athletes reported widespread cortical thinning in frontal and bilateral temporoparietal cortices associated with post-concussive symptoms (Albaugh et al., 2015).

Between-group analysis showed significant differences in two clusters (left parietal and left temporal), with patients displaying reduced area. When considering the subsample of 30 patients reporting moderate-to-severe symptoms and their 30 HC, five of six clusters demonstrated area reductions between the two groups. Hence, focusing on the subgroup of patients with more severe symptoms may have increased area diminutions compared with HC. Other studies have devoted attention to the initial post-injury periods, but found no group differences in volume or thickness (Ling et al., 2013; Toth et al., 2013; Narayana et al., 2015). As suggested from these examinations, measurable atrophy due to neuronal loss may occur later in the post-injury course of mTBI. In fact, as reported by Mayer et al. (2015), signs of cortical atrophy were demonstrated over a 4-month study interval in mTBI children compared to controls (Mayer et al., 2015). The location of the observed bilateral frontal cluster is in line with our results.

In addition, morphometric studies concentrating on chronic effects of mTBI have provided evidence for progressive morphological alterations and more accentuated damage of GM integrity (Tremblay et al., 2013; Zhou et al., 2013; Tate et al., 2014).

For instance, significant decreases in brain volume have been demonstrated after a single mTBI over the first 13 months in the anterior cingulate gyrus (bilateral), left cingulate gyrus isthmus, and right precuneus (Zhou et al., 2013). Briefly, all these studies detected thickness or volume loss long after injury, leading us to speculate that area reduction might be specific to the acute period and that changes in cortical thickness might be observed later in the course of mTBI. As pathological interpretation of area reduction remains unclear, we can only conjecture possible explanations for our results. Tissue deformation in the form of area contraction could be a direct result of different forces acting on the brain tissue including angular acceleration (Feng et al., 2010; Hansen et al., 2013). In conclusion, a simultaneous but separate evaluation of cortical surface area, thickness, and volume is important to provide more comprehensive information about the underlying pathogenic changes following mTBI. To date, we are not aware of any morphometric mTBI studies that have considered the examination of area as a distinct cortical feature.

### Convergence of the white and gray matter findings

Patients reporting higher levels of complaints within the first week following mTBI showed a concomitant reduction of structural connectivity and cortical surface area. No significant relationships between symptom severity and structural alterations could be found in the HC, suggesting that WM and GM anomalies observed in the patients reflect injury-specific alterations. Moreover, the inverse correlations between both structural MRI measures and symptom severity together with the observed anatomical overlap clearly emphasize the clinical significance of the present findings. Given the availability of only one scan, it remains unclear whether reduced WM integrity is the trigger for area reductions seen in anatomically connected areas or vice versa. The findings reported for the frontal regions are in agreement with many mTBI studies regarding structural and functional alterations discussed in a recent meta-analysis (Eierud et al., 2014). Furthermore, the frontal cortex is among the brain regions identified as being more biomechanically vulnerable to impact forces (McAllister and Stein, 2010; Zappala et al., 2012; Wright et al., 2013). Temporal cortices, corpus callosum and subcortical structures are also regions susceptible to injury in mTBI (Viano et al., 2009; Bigler and Maxwell, 2012). Moreover, the occurrence of DAI is frequently located within the frontal lobes, as revealed by various epidemiological studies (Niogi et al., 2008; Chatelin et al., 2011). The structural WM/GM changes in the right PoCG are difficult to explain. Perhaps the primary somatosensory cortex is associated with headaches frequently reported by mTBI patients or with the experience of pain (Gosselin et al., 2012). Nevertheless, another study also observed an inverse correlation between cortical thickness in bilateral temporoparietal regions and post-concussive symptoms in the subacute post-injury phase (Albaugh et al., 2015). Another finding of the present study is the involvement of the insula, which showed decreased connectivity within the dysconnected subnetwork and decreased cortical surface area using the ROI approach. The insula is an important hub for monitoring, switching, attention, mental flexibility, error processing, cognitive control and is a key region involved in all subjective feelings from the body including pain (Menon et al., 2001; Sridharan et al., 2008; Craig, 2009; Ham et al., 2014; Wiebking et al., 2014). Since individuals with mTBI complain for example of poor concentration and greater irritability, damage to the insula's GM and/or its connectivity might have the potential to explain such conditions. Recent studies with resting-state fMRI, arterial spin labeling MRI and magnetoencephalography have also revealed a responsibility of the insula in early stage post-mTBI (da Costa et al., 2015; Meier et al., 2015; Zhan et al., 2015).

### Correlations between structural brain alterations and cognitive performance

The extent to which WM/GM alterations linked to symptom severity is associated with executive-attentive functions was explored in the patient group. Only area reduction in the bilateral frontal clusters was related to poor performance in the Go/Nogo task, a paradigm commonly employed to study selective attention and response inhibition. Performance in the Go/Nogo task has been found to be particularly dependent on the functioning of the ACC (Botvinick et al., 2004), dorsolateral PFC (MacDonald et al., 2000), right inferior and superior frontal gyrus (Aron et al., 2004; Floden and Stuss, 2006), OFC (Eagle et al., 2008), striatum (Vink et al., 2005), and on frontostriatal connectivity (Liston et al., 2006). The majority of these structures has been shown to be altered in the present study.

### Limitations

Several limitations of the present study are worth mentioning. First, the sample of patients investigated here is heterogeneous with respect to localization of impact and mechanism of injury. Nevertheless, the advantages of our carefully selected sample of patients include its large size, the absence of parenchymal abnormalities visible on CT and MRI, and the strict data collection times, which minimize the confounding effect of the dynamic post-injury process. Second, several symptoms listed in the RPQ are not specific to mTBI since these complaints are also experienced by the general population (Chan, 2001; Garden et al., 2010). In fact, 50.9% of the HC in our study perceived at least one symptom. Despite the overlap in RPQ score across the two groups, we did not exclude either non-suffering mTBI participants or symptoms-reporting HC, as these individuals represented a significant proportion of their groups. This fact might limit the specificity of the findings, although no correlates of symptoms severity in the brain structures of the HC were detected. Third, it is critical to note that differences between groups in cortical surface area were found based on the results of the correlation with symptoms severity, i.e., only the detected clusters were further investigated. This could have important implications for future groups, as they would need to replicate the findings of the correlation analyses prior to compare against the HC.

Finally, it is important to note that the DTI model has many limitations. Specifically, the tensor model is not able to detect heterogeneous orientations of fiber bundles within a single voxel (Mori and van Zijl, 2002) and is known to be susceptible to noise (Zalesky and Fornito, 2009), noise for which a Gaussian distribution has been assumed. To resolve e.g., the fiber-crossing problem, diffusion spectrum MRI (Wedeen et al., 2008) and related promising methods (Tuch, 2004; Hess et al., 2006) were developed capable to map complex fiber trajectories and to circumvent potentially bias findings. Furthermore, although it has been recommended to acquire several *b* = 0 images and average them in order to increase the signal-to-noise ratio (SNR), we acquired only one *b* = 0 image. However, this *b* = 0 image has a sufficiently high SNR. Nevertheless, the DTI model is capable to provide useful information about structural connectivity in the living human brain. With respect to surface-based morphometry, the procedures for measuring cortical thickness as implemented in FreeSurfer have been validated against histological analysis (Rosas et al., 2002) and manual measurements (Kuperberg et al., 2003; Salat et al., 2004). With respect to cortical surface area, however, validation against histological analysis is still missing in the literature, although the methodology is well developed and described (Winkler et al., 2012).

Important directions for future multimodal investigations include the additional integration of longitudinal and functional brain connectivity measures. Ultimately, tracking the specific structural pattern of patients with symptomatic mTBI as the clinical population of interest will help to further distinguish the anomal progression or recovery and may help improving diagnostic classification. Our research group is currently addressing structural longitudinal investigations combined with functional MRI approach in order to provide potential predictive markers of a positive or adverse outcome after mTBI.

## Conclusions

For the first time, we show that the combination of DTI-based network analyses and T1-weighted SBM measures provides essential information about clinical changes, clearly complementary to classical DTI and voxel-based morphometry markers of mTBI. Both decreased connectivity and reduction in surface area were inversely associated with subjective complaints resulting from acute mTBI, especially in frontal brain structures and in the insula. We show that mTBI leads to structural changes already during the first week following injury and that these damages might be greater in the acute period than previously believed. This study also provides evidence that mTBI affects the integrity of WM networks encouraging the assumption of a dysconnection syndrome.

Whether these early biomarkers have predictive value needs to be tested in longitudinal studies. Finally, the findings suggest that the rapid identification of high-risk patients with the use of clinical scales should be assessed acutely in the medical setting.

## Author contributions

PD contributed to the design of the study, coordinated the study, clinically evaluated the patients, conducted the statistical analyses, interpreted the data for the study and drafted the manuscript. SJ designed and conceptualized the study, obtained funding, interpreted the data for the study, supervised the study and critically revised the manuscript. LM contributed to the acquisition of data (patients' recruitment) and critically revised the manuscript. HS contributed to the acquisition of data (patients' recruitment) and critically revised the manuscript. RG contributed to the acquisition of data (patients' recruitment) and critically revised the manuscript. JF contributed to the acquisition of data (patients' recruitment) and critically revised the manuscript. MS contributed to the acquisition of data (patients' recruitment) and critically revised the manuscript. CM contributed to the acquisition of data (patients' recruitment) and critically revised the manuscript. EU analyzed the neuroradiological data and critically revised the manuscript. AM designed and conceptualized the study, obtained funding and critically revised the manuscript. LJ contributed to the design of the study, interpreted data for the study and critically revised the manuscript. JH contributed to the acquisition of MRI data, contributed to statistical analyses, analyzed and interpreted data for the study, drafted the manuscript, supervised the study and critically revised the manuscript. All authors gave final approval of the version to be published and gave agreement to be accountable for all aspects of the work.

## Funding

This work has been supported by the Research Fund of the Swiss Accident Insurance (SUVA).

### Conflict of interest statement

The authors declare that the research was conducted in the absence of any commercial or financial relationships that could be construed as a potential conflict of interest. SJ was the PI of the study and is medical director of the Bellikon Rehabilitation Clinic, owned by SUVA. PD received financial support from SUVA. The views expressed in the present manuscript are those of the authors and not necessarily those of the SUVA.

## Abbreviations

DAI: diffuse axonal injuries
DTI: diffusion tensor imaging
GCS: Glasgow coma scale
GM: gray matter
HC: healthy controls
mTBI: mild traumatic brain injury
NBS: network-based statistic
RPQ: Rivermead Post Concussion Symptoms Questionnaire
SBM: surface-based morphometric
WM: white matter.
