# Supplementary material for: Connectomic and Surface-Based Morphometric Correlates of Acute Mild Traumatic Brain Injury
Source: Front Hum Neurosci. 2016 Mar 29;10:127. doi: 10.3389/fnhum.2016.00127 (PMC4809899; doi:10.3389/fnhum.2016.00127)
Supplement: Supplementary file 4 [file DataSheet1.docx]

***SUPPLEMENTARY MATERIAL***

**Connectomic and surface-based morphometric correlates of acute mild traumatic brain injury**

Patrizia Dall’Acqua^*^, Sönke Johannes, Ladislav Mica, Hans-Peter Simmen, Richard Glaab, Javier Fandino, Markus Schwendinger, Christoph Meier, Erika Jasmin Ulbrich, Andreas Müller, Lutz Jäncke and Jürgen Hänggi

*Correspondence: Patrizia Dall’Acqua: patrizia.dallacqua@rehabellikon.ch

**Supplementary Data**

**Neuropsychological assessment**

The neuropsychological investigation focusing on the domains of attention, executive and memory functions included the following tests: (i) subtests Alertness (intrinsic and phasic), Go/Nogo 1 from 2, and Divided attention of the Test for Attentional Performance (TAP 2.2) ([Zimmermann and Fimm, 2002](#_ENREF_26)) to measure attention, inhibitory control, and cognitive flexibility with varying complexity; (ii) German version ([Von Aster et al., 2006](#_ENREF_23)) of the Backward Digit Span of the Wechsler Adult Intelligence Scale WAIS-III ([Wechsler, 1997](#_ENREF_24)) to assess verbal working memory and the Swiss adaptation ([Balzer et al., 2011](#_ENREF_1)) of the word generation fluency task ([Benton et al., 1994](#_ENREF_3)) to measure the ability of planning, of selecting verbal production, and of control processes; (iii) Swiss adaptation ([Balzer et al., 2011](#_ENREF_1)) of the Rey Auditory Verbal Learning Tests RAVLT ([Strauss et al., 2006](#_ENREF_21)) to assess verbal learning (using the total number of words recalled across five trials) and short- and long-delay verbal recall and Swiss adaptation ([Balzer et al., 2011](#_ENREF_1)) of the Rey/Taylor Complex Figure Test RTCFT ([Rey, 1959](#_ENREF_16);[Strauss et al., 2006](#_ENREF_21)) to measure delayed figural recall. In addition, an estimation of nonverbal intelligence level was performed using the Wiener Matrizen-Test 2 WMT-2 ([Formann et al., 2011](#_ENREF_12)), an adapted version based on Raven’s progressive matrix test ([Raven, 1958](#_ENREF_15)).

**Magnetic resonance imaging data acquisition**

MRI scans were acquired on a 3.0 Tesla Philips Ingenia whole body scanner (Philips Medical Systems, Best, The Netherlands) equipped with a transmit-receive body coil and a commercial 15-elements transmit-receive head coil array that is capable of sensitivity encoding (SENSE).

A diffusion-weighted spin echo echo-planar imaging (EPI) sequence was used to obtain diffusion-weighted scans with a measured and reconstructed spatial resolution of 2.0 x 2.0 x 2.0 mm^3^ (acquisition and reconstruction matrix 112 x 112 pixels, 75 slices with a thickness of 2 mm, no gap between slices). Further imaging parameters were: field of view (FOV) = 224 x 224 mm^2^; echo time (TE) = 64.90 ms; repetition time (TR) = 18.714 s; α = 90°; SENSE factor R = 2.1; b-value b = 1,000 s/mm^2^; and number of averages = 1, acquisition time (min) 23:05. Diffusion was measured along 64 non-collinear directions preceded by a non-diffusion-weighted volume (reference volume).

A T1-weighted fast field echo (FFE) sequence was used to map the B_0_ field in order to correct the DTI data for EPI-related geometrical distortions. The B_0_ map (3D echo sequence) is composed of a magnitude and a phase image and was measured with a spatial resolution of 2.0 x 2.0 x 4.0 mm^3^ (acquisition matrix 112 × 56 pixels, 75 slices) and reconstructed to a spatial resolution of 2.0 x 2.0 x 2.0 mm^3^ (acquisition matrix 112 × 112 pixels, 75 slices). Further imaging parameters were: FOV = 224 × 224 mm^2^, 75 slices, dual echo-time TE = 3.60 / 5.63 ms, repetition-time TR = 30.0 ms, flip-angle α = 60°, acquisition time (min) 4:11.

A volumetric 3D T1-weighted gradient echo sequence (turbo field echo) image was measured with a spatial resolution of 1.0 × 1.0 × 1.0 mm^3^ (acquisition matrix 240 × 240 pixels, 160 sagittal slices) and reconstructed to a spatial resolution of 0.94 × 0.94 × 1.0 mm^3^ (reconstruction matrix 256 × 256 pixels, 160 sagittal slices). Further imaging parameters were: FOV = 240 × 240 mm^2^, TE = 3.70 ms, TR = 8.14 ms, flip-angle α = 8°, SENSE factor R = 1.8, acquisition time (min) 7:29.

Beside these three MRI sequences, resting-state functional MRI and susceptibility weighted imaging were also applied, but these data are not part of the present manuscript. MRI scans such as T2-weighted, proton-density-weighted, and fluid attenuated inversion recovery were additionally acquired in order to exclude brain anomalies. All T1-weighted and conventional MRI scans were evaluated by the same radiologist (E.J.U.) and in cases of any salient features the subjects were excluded from the sample.

**Preprocessing of diffusion tensor imaging data**

Preprocessing of the diffusion-weighted MRI data was performed with FSL tools (FMRIB software library; version 5.0.6; http://www.fmrib.ox.ac.uk/fsl/) ([Smith et al., 2004](#_ENREF_20)) such as the FDT (FMRIB diffusion toolbox; version 3.0) ([Behrens et al., 2003](#_ENREF_2)). For deterministic fibre tractography we used the Diffusion Toolkit (DTK, version 0.6.2.1) and TrackVis software (version 0.5.2.1; http://trackvis.org/) ([Park et al., 2009](#_ENREF_14)). The connectivity matrix was computed in MATLAB (version 8.0.0.783; <http://www.mathworks.com/index.html>). To construct the connectivity matrix of the WM pathways, the following fully automated preprocessing steps were realized: 1) In a first step, a binary brain mask was created using FSL’s brain extraction tool (BET). This mask is used in later steps to exclude non-brain tissue. 2) Eddy current distortions and head movements were corrected by applying the EDDY_CORRECT tool of FDT using one b=0 image as reference image. 3) EPI-related geometrical distortions due to magnetic field inhomogeneities were unwarped using the B_0_ map and FSL’s FUGUE tool. 4) Diffusion gradients were adjusted for rotations introduced by the eddy current and head movement corrections. 5) The preprocessed DTI data were then subjected to the DTK to compute voxel-wise diffusion tensors and to construct the (principal) eigenvector and eigenvalue maps as well as a map of fractional anisotropy (FA). 6) Deterministic tractography was conducted in TrackVis using the “brute force” approach with an interpolated streamline tracking algorithm. Twenty streamlines per voxel were propagated and fibre tracking was stopped if FA was lower than 0.10 or if the turning angle of a streamline between two consecutive voxels was larger than 45°. This resulted in a whole brain connectome comprised by about 2-3 millions of streamlines including subcortical pathways and connections to the cerebellum. 7) The individual FA map was registered onto the FMRIB58-FA template, which is in correspondence with the MNI152 standard space, using FSL’s linear image registration tool (FLIRT) and the resulting transformations were stored. Due to the nature of the streamlines we run FLIRT and not FNIRT. Applying nonlinear transformations can result in „tearing“ streamlines (and in addition, longer streamlines are more prone to tear) so that when computing the connectivity measure (number of streamlines between two brain regions in our study) „teared“ streamlines are not counted any longer.

8) These transformations were then applied to the streamlines produced in step 6 in order to transform the streamlines into the MNI152 space. It is important to note that spatial normalization into standard space has been applied after tractography has been performed in native space, i.e. only the resulting streamlines (reconstructed white matter fibres) were transformed, not the preprocessed DTI images that are used for tractography. 9) The automated anatomical labeling (AAL) regions of interest (ROIs) ([Tzourio-Mazoyer et al., 2002](#_ENREF_22)), which are already in MNI152 standard space, were used to count the number of streamlines between each pair of ROIs. This AAL template consists of 90 ROIs (45 in each hemisphere) covering the entire neocortex (78 cortical ROIs) as well as the subcortical structures amygdala, hippocampus, thalamus, caudate, putamen, and pallidum (12 subcortical ROIs). 10) Streamlines connected to the cerebellum, those running through the brainstem, and streamlines shorter than 5 mm in length were removed (denoted streamlines omitted). Streamlines that make connections within a ROI itself were deleted (denoted selfloops). The number of the remaining streamlines between any pair of ROIs (denoted streamlines used to populate matrix) was counted using MATLAB scripts ([Zalesky et al., 2010](#_ENREF_25)). 11) This procedure resulted in an undirected, weighted 90 x 90 (45 nodes per hemisphere) connectivity matrix for each individual participant. The strength of a structural connection was operationalized by the number of reconstructed streamlines between two ROIs. 12) The undirected, weighted 90 x 90 nodes connectivity matrices were then subjected to a network-based statistical analysis (see below).

**Preprocessing of surface-based morphometry data**

Cortical surface reconstruction was performed with the FreeSurfer image analysis suite (version 5.3.0), which is documented and freely available online (<http://surfer.nmr.mgh.harvard.edu/>). The technical details of these procedures are described in prior publications ([Fischl et al., 1999a](#_ENREF_9);[Fischl et al., 1999b](#_ENREF_10);[Fischl and Dale, 2000](#_ENREF_7);[Fischl et al., 2004a](#_ENREF_8);[Fischl et al., 2004b](#_ENREF_11)). The 3D structural high resolution T1-weighted MRI scan was used to construct models of each subject’s cortical surface in order to measure cortical thickness and surface area. This fully automated procedure comprised segmentation of the cortical and subcortical WM ([Dale et al., 1999](#_ENREF_4)), tessellation of the GM/WM boundary, inflation of the folded surface tessellation patterns ([Fischl et al., 1999a](#_ENREF_9);[Fischl et al., 1999b](#_ENREF_10)) and automatic correction of topological defects in the resulting manifold ([Fischl and Dale, 2000](#_ENREF_7)). This cortical surface was then used as starting point for a deformable surface algorithm designed to find the grey/white and pial (GM/cerebrospinal fluid) interfaces with sub-millimetre precision ([Fischl and Dale, 2000](#_ENREF_7)). The procedures for measuring cortical thickness have been validated against histological analysis ([Rosas et al., 2002](#_ENREF_17)) and manual measurements ([Kuperberg et al., 2003](#_ENREF_13);[Salat et al., 2004](#_ENREF_19)). This method uses both intensity and continuity information from the surfaces in the deformation procedure in order to interpolate surface locations for regions in which the MRI scan is ambiguous ([Fischl and Dale, 2000](#_ENREF_7)). For each subject, cortical surface area, thickness and volume of the cortical ribbon was computed on a uniform grid (comprised by vertices) with about 1 mm spacing across both cortical hemispheres, with the thickness being defined by the shortest distance between the grey/white and pial surface models. The thickness maps produced are not limited to the voxel resolution of the image and thus sensitive for sub-millimetre differences between groups ([Fischl and Dale, 2000](#_ENREF_7)). The way in which the resolution of the cortical thickness maps goes beyond the resolution of the original acquisition is conceptually similar to a (conventional) partial volume correction procedure. The cortex is smooth at the spatial scale of a several millimetres, which is imposed as constraint by FreeSurfer to estimate the location of the surface with subvoxel accuracy. For instance, if a given voxel is darker than its neighbouring GM it probably contains more cerebrospinal fluid and so the surface model is at a slightly different position than if the neighbouring voxels were brighter and therefore contain probably more WM. Cortical surface area, thickness and volume measures were mapped onto the inflated surface of each participant’s brain reconstruction, this allowing visualization of data across the entire cortical surface (gyri and sulci) without the data being obscured by cortical folding. Data were re-sampled for all subjects and rendered onto a common spherical coordinate system ([Fischl et al., 1999b](#_ENREF_10)). Then surface-based, vertex-wise cortical surface area, thickness and volume maps were computed for each participant. For the whole-brain vertex-wise analysis, the data were smoothed on the surface tessellation using an iterative nearest-neighbour averaging procedure with 166 iterations on the left hemisphere and 167 iterations on the right hemisphere, corresponding to a 2D surface-based diffusion-smoothing kernel with a full width at half maximum of about 15 mm. These cortical surface area, thickness, and volume maps were then subjected to statistical analyses using statistical tools implemented in FreeSurfer (see below).

In addition, the cerebral cortex was parcellated into units based on gyral/sulcal structure as implemented in FreeSurfer ([Desikan et al., 2006](#_ENREF_5);[Destrieux et al., 2010](#_ENREF_6)). Quality assessment was done using FreeSurfer’s quality assessment (QA) tools (<https://surfer.nmr.mgh.harvard.edu/fswiki/QATools>). Reconstructed surface models were visually checked for accuracy by an experienced imaging analyst (J.H.) and no manual interventions were necessary.

**Supplementary Figures and Table**

**Supplementary Figures**

**Supplementary Figure 1: Inverse correlation between surface area and total Rivermead Post Concussion Symptoms Questionnaire score over both groups (n = 104)**

**
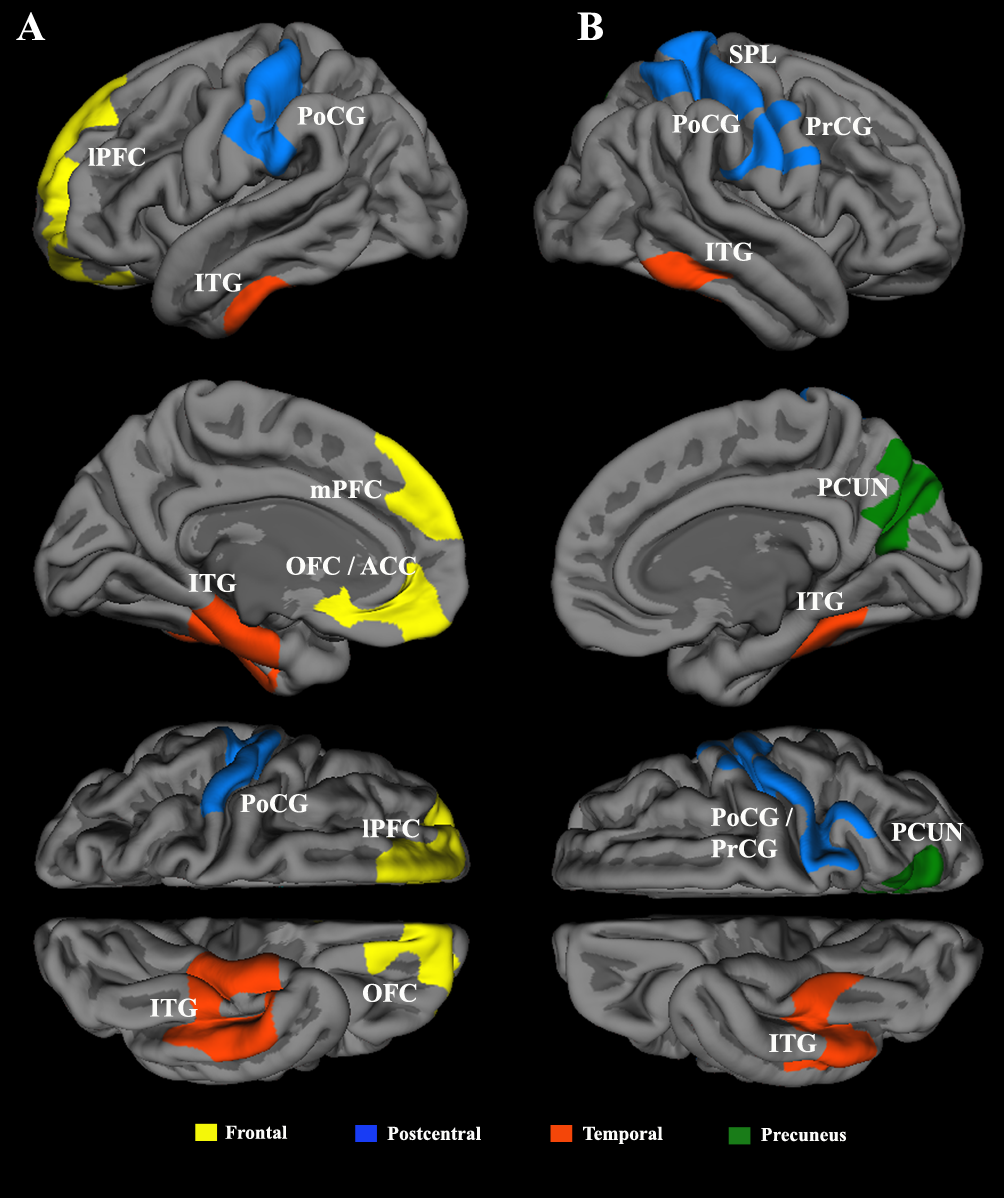
**

Panels of the top row show the lateral hemispheres (A = left, B = right), whereas the second row shows the medial hemispheres. The third and bottom rows represent the superior and inferior views, respectively. Only clusters exceeding a cluster-wise corrected probability of p<0.05 are shown.

**Supplementary Figure 2: Group differences in surface area (51 patients / 53 controls)**


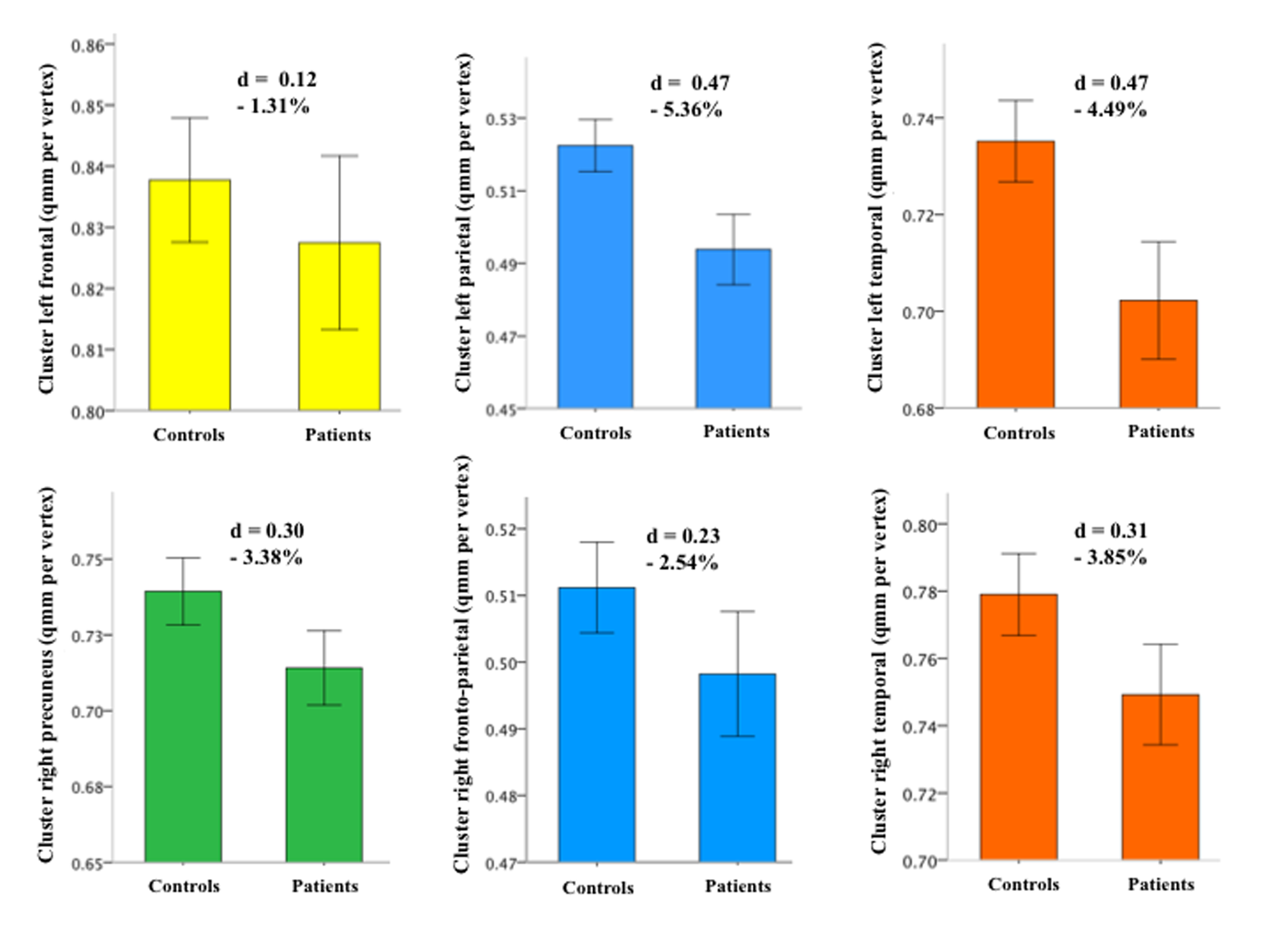


The same colours used in Supplementary Figure 1 for the cortical clusters were assigned here for the bar charts. Depending on the cluster, the percentage of surface area reduction in the patient group varied between 1.3% and 5.4%. [Error bar = ± standard error of mean SEM; the strength of the group difference (i.e. effect size) was assessed using Cohen’s d score.].

**Supplementary Table**

**Supplementary Table 1: Neuropsychological assessment results by group**

|  | **Patients  (n = 51)** | | | | **Controls  (n = 53)** | | | |  |
| --- | --- | --- | --- | --- | --- | --- | --- | --- | --- |
| **Neuropsychological assessment** | **Mean** | **SD** | **Range (Min. / Max.)** | | **Mean** | **SD** | **Range (Min. / Max.)** | | **p value (1-sided)^e^** |
| BDI-II^a^ | 6.37 | 5.7 | 0 | 35 | 3.81 | 4.5 | 0 | 15 | 0.013 |
| BAI^b^ | 2.71 | 6.5 | 0 | 32 | 0.72 | 2.3 | 0 | 11 | 0.043 |
| Intellectual ability (IQ) | 100.90 | 15.0 | 73 | 126 | 107.51 | 14.3 | 73 | 126 | 0.024 |
| Alertness, tonic (ms) | 242.53 | 52.5 | 186 | 449 | 219.28 | 20.3 | 181 | 285 | 0.020 |
| Alertness, phasic (ms) | 244.06 | 69.0 | 189 | 602 | 219.87 | 22.8 | 180 | 323 | 0.010 |
| Go/Nogo (errors) | 0.90 | 1.2 | 0 | 5 | 1.02 | 1.1 | 0 | 4 | 0.303 |
| Divided attention, auditory (ms) | 585.35 | 92.8 | 378 | 945 | 559.26 | 76.3 | 401 | 715 | 0.060 |
| Divided attention (omissions) | 1.09 | 1.4 | 0 | 6 | 1 | 1.1 | 0 | 4 | 0.348 |
| Word fluency (total score) | 27.55 | 6.8 | 11 | 42 | 32.24 | 8.3 | 15 | 49 | 0.010 |
| Word fluency (error percent) | 5.20 | 5.5 | 0 | 21.43 | 4.23 | 4.4 | 0 | 16.70 | 0.159 |
| Working memory (length) | 5.33 | 1.2 | 3 | 7 | 5.83 | 1.3 | 3 | 8 | 0.020 |
| AVLGT^c^ recall score (words) | 57.25 | 7.1 | 41 | 72 | 59.34 | 6.9 | 38 | 71 | 0.066 |
| AVLGT short delayed score (words) | 12.72 | 1.9 | 8 | 15 | 13.13 | 2.0 | 7 | 15 | 0.151 |
| AVLGT long delayed score (words) | 12.98 | 1.8 | 7 | 15 | 13.25 | 1.9 | 8 | 15 | 0.231 |
| RTCFT^d^ long delayed recall (figure) | 11.92 | 2.4 | 7 | 17 | 12.96 | 2.5 | 5.5 | 18 | 0.017 |
| RTCFT displaced recall (figure) | 1.44 | 1.1 | 0 | 4 | 1.16 | 1.3 | 0 | 4 | 0.114 |
| RTCFT deformed recall (figure) | 1.39 | 0.8 | 0 | 4 | 1.34 | 0.9 | 0 | 4 | 0.375 |
| ^a^ BDI-II = Beck Depression Inventory, 2nd edition; ^b^ BAI = Beck Anxiety Inventar; ^c^ AVLGT = German adaptation of the Rey Auditory Verbal Learning Tests RAVLT; ^d^ RTCFT = Rey/Taylor Complex Figure Test.  ^e^ Based on studies reporting poorer cognitive performance in the initial phase after mTBI compared to controls ([Rosenbaum and Lipton, 2012](#_ENREF_18)), differences between groups in neuropsychological assessment were analyzed using one-tailed t-tests. | | | | | | | | | |

**Supplementary Table 2: Edges list of the correlation between structural connectivity and total Rivermead Post Concussion Symptoms Questionnaire score in the patient group**

| 50 grey-matter nodes and 59 inter-regional connections | | T-value | Pearson's correlation coefficient (df = 48) |
| --- | --- | --- | --- |
| Frontal_Inf_Orb_R | Insula_R. | 3.94 | -0.49 |
| Insula_L | ParaHippocampal_L. | 3.76 | -0.48 |
| Insula_L | Temporal_Inf_L. | 3.04 | -0.40 |
| Supp_Motor_Area_L | Insula_L. | 2.96 | -0.39 |
| Temporal_Sup_L | Temporal_Mid_L. | 2.89 | -0.38 |
| Precentral_L | Frontal_Inf_Oper_L. | 2.87 | -0.38 |
| Olfactory_L | Insula_L. | 2.81 | -0.38 |
| Insula_R | ParaHippocampal_R. | 2.75 | -0.37 |
| Supp_Motor_Area_L | Cingulum_Mid_R. | 2.72 | -0.37 |
| Insula_L | Angular_L | 2.65 | -0.36 |
| Frontal_Inf_Oper_L | Rolandic_Oper_L. | 2.62 | -0.35 |
| Supp_Motor_Area_L | Caudate_L. | 2.62 | -0.35 |
| Frontal_Inf_Orb_L | Cingulum_Ant_L. | 2.61 | -0.35 |
| Frontal_Inf_Tri_L | Cingulum_Mid_L. | 2.52 | -0.34 |
| Temporal_Sup_L | Temporal_Inf_L. | 2.52 | -0.34 |
| Supp_Motor_Area_L | Cingulum_Ant_L. | 2.46 | -0.33 |
| Insula_R | Hippocampus_R. | 2.46 | -0.33 |
| Precentral_R | Frontal_Inf_Oper_R. | 2.41 | -0.33 |
| Precentral_L | Frontal_Inf_Tri_L. | 2.38 | -0.32 |
| SupraMarginal_R | Temporal_Sup_R. | 2.38 | -0.32 |
| Frontal_Sup_Orb_L | Frontal_Inf_Orb_L. | 2.32 | -0.32 |
| Frontal_Sup_Medial_L | Cingulum_Mid_L. | 2.31 | -0.32 |
| Frontal_Inf_Oper_R | Insula_R. | 2.3 | -0.32 |
| Cingulum_Ant_L | Hippocampus_L. | 2.25 | -0.31 |
| Frontal_Sup_R | Cingulum_Ant_R. | 2.24 | -0.31 |
| Frontal_Mid_R | Frontal_Inf_Tri_R. | 2.23 | -0.31 |
| ParaHippocampal_L | Temporal_Pole_Sup_L. | 2.22 | -0.31 |
| Frontal_Mid_L | Insula_L. | 2.19 | -0.30 |
| Caudate_L | Thalamus_L. | 2.19 | -0.30 |
| Frontal_Sup_Orb_L | Frontal_Mid_Orb_L. | 2.18 | -0.30 |
| Frontal_Med_Orb_L | Cingulum_Ant_L. | 2.17 | -0.30 |
| Frontal_Sup_Orb_L | Rectus_L. | 2.14 | -0.30 |
| Caudate_L | Pallidum_L. | 2.14 | -0.30 |
| Fusiform_R | Temporal_Sup_R. | 2.14 | -0.30 |
| Frontal_Sup_Orb_L | Olfactory_L. | 2.06 | -0.29 |
| Frontal_Inf_Orb_L | Insula_L. | 2.06 | -0.29 |
| Fusiform_R | Temporal_Pole_Sup_R. | 2.06 | -0.29 |
| Cingulum_Mid_L | Cingulum_Mid_R. | 2.03 | -0.28 |
| Frontal_Inf_Oper_L | Temporal_Pole_Sup_L. | 2.01 | -0.28 |
| Caudate_L | Cingulum_Ant_R. | 2.01 | -0.28 |
| Frontal_Inf_Orb_L | Rolandic_Oper_L. | 1.98 | -0.27 |
| Insula_L | Cingulum_Ant_L. | 1.98 | -0.27 |
| Frontal_Mid_R | Heschl_R. | 1.98 | -0.27 |
| ParaHippocampal_R | Thalamus_R. | 1.97 | -0.27 |
| Supp_Motor_Area_L | Supp_Motor_Area_R. | 1.96 | -0.27 |
| Frontal_Inf_Orb_R | Heschl_R. | 1.96 | -0.27 |
| ParaHippocampal_R | SupraMarginal_R. | 1.93 | -0.27 |
| Frontal_Sup_L | Frontal_Sup_Medial_L. | 1.9 | -0.26 |
| Caudate_R | Thalamus_R. | 1.9 | -0.26 |
| Rolandic_Oper_L | Supp_Motor_Area_L. | 1.89 | -0.26 |
| Frontal_Sup_R | Frontal_Sup_Medial_R. | 1.88 | -0.26 |
| Precentral_R | Precuneus_R. | 1.88 | -0.26 |
| Frontal_Inf_Oper_R | Fusiform_R. | 1.87 | -0.26 |
| Cingulum_Ant_L | Precentral_R. | 1.86 | -0.26 |
| Insula_R | Fusiform_R. | 1.86 | -0.26 |
| Precentral_L | Postcentral_R. | 1.86 | -0.26 |
| Insula_R | Postcentral_R. | 1.85 | -0.26 |
| Frontal_Inf_Orb_R | Rolandic_Oper_R. | 1.81 | -0.25 |
| Rolandic_Oper_R | Insula_R. | 1.8 | -0.25 |

**Abbreviation can be found in Tzourio et al. (**[**Tzourio-Mazoyer et al., 2002**](#_ENREF_22)**)**

**Supplementary Table 3: Edges list of group difference in the structural connectivity between subsamples (30 mTBI patients < 30 matched controls)**

| 48 grey-matter nodes and 57 inter-regional connections | | T-value |
| --- | --- | --- |
| Frontal_Inf_Oper_L | Rolandic_Oper_L. | 3.19 |
| Precentral_L | Rolandic_Oper_L. | 2.78 |
| Precentral_L | Frontal_Inf_Tri_L. | 2.74 |
| Frontal_Inf_Orb_R | Insula_R. | 2.69 |
| Insula_L | Temporal_Inf_L. | 2.67 |
| Supp_Motor_Area_R | Caudate_R. | 2.63 |
| Frontal_Inf_Oper_L | Frontal_Inf_Tri_L. | 2.61 |
| Paracentral_Lobule_L | Temporal_Inf_L. | 2.47 |
| Precentral_R | Occipital_Mid_R. | 2.46 |
| Frontal_Inf_Oper_L | Temporal_Sup_L. | 2.45 |
| Frontal_Inf_Orb_R | Fusiform_R. | 2.45 |
| SupraMarginal_R | Precuneus_R. | 2.43 |
| Temporal_Pole_Sup_R | Temporal_Mid_R. | 2.43 |
| Rolandic_Oper_R | Insula_R. | 2.33 |
| Temporal_Pole_Sup_L | Temporal_Mid_L. | 2.28 |
| Paracentral_Lobule_L | Temporal_Mid_L. | 2.25 |
| Precuneus_L | Temporal_Inf_L. | 2.24 |
| Caudate_R | Putamen_R. | 2.24 |
| Occipital_Mid_R | Parietal_Sup_R. | 2.23 |
| Frontal_Inf_Oper_L | Temporal_Inf_L. | 2.22 |
| Cingulum_Mid_L | Paracentral_Lobule_L. | 2.19 |
| Postcentral_L | Precuneus_L. | 2.16 |
| Fusiform_R | Temporal_Pole_Sup_R. | 2.16 |
| Supp_Motor_Area_R | Insula_R. | 2.15 |
| Insula_R | Caudate_R. | 2.15 |
| Precentral_R | Frontal_Inf_Oper_R. | 2.13 |
| Hippocampus_L | Paracentral_Lobule_L. | 2.12 |
| Frontal_Sup_Medial_L | Pallidum_L. | 2.10 |
| Cuneus_R | Parietal_Sup_R. | 2.10 |
| Temporal_Sup_R | Temporal_Inf_R. | 2.10 |
| Precentral_R | SupraMarginal_R. | 2.06 |
| Insula_L | Amygdala_L. | 2.05 |
| Cingulum_Post_L | Fusiform_L. | 2.04 |
| Occipital_Inf_L | Temporal_Inf_L. | 2.03 |
| Precentral_R | Lingual_R. | 2.03 |
| Occipital_Sup_R | Occipital_Mid_R. | 2.01 |
| Frontal_Mid_L | Insula_L. | 1.98 |
| Cuneus_R | Occipital_Sup_R. | 1.98 |
| Frontal_Inf_Oper_R | Fusiform_R. | 1.98 |
| Cingulum_Ant_L | Caudate_R. | 1.96 |
| Precentral_R | Putamen_R. | 1.95 |
| Parietal_Inf_R | Temporal_Mid_R. | 1.94 |
| Paracentral_Lobule_R | Temporal_Inf_R. | 1.94 |
| Amygdala_L | Fusiform_L. | 1.92 |
| Temporal_Sup_L | Temporal_Inf_L. | 1.92 |
| Frontal_Inf_Orb_R | Temporal_Mid_R. | 1.91 |
| Temporal_Mid_R | Temporal_Inf_R. | 1.91 |
| Frontal_Inf_Orb_L | Insula_L. | 1.90 |
| Paracentral_Lobule_L | Supp_Motor_Area_R. | 1.89 |
| Frontal_Sup_L | Temporal_Sup_L. | 1.88 |
| Precentral_R | Cingulum_Ant_R. | 1.87 |
| Lingual_R | Temporal_Mid_R. | 1.87 |
| Paracentral_Lobule_R | Temporal_Mid_R. | 1.87 |
| Frontal_Mid_Orb_L | Insula_L. | 1.86 |
| Paracentral_Lobule_L | Pallidum_L. | 1.86 |
| Frontal_Inf_Oper_R | Supp_Motor_Area_R. | 1.86 |
| Cuneus_R | Parietal_Inf_R. | 1.85 |

**Supplementary Table 4: Inverse correlation between surface area and total RPQ score over both groups (n = 104)**

| **Measure and anatomical location** | **Cluster name** | **Cluster color in Supplem. Fig. 1** | **Cluster size (mm^2^)** | **Number of vertices** | **MNI coordinates   (x, y, z)** | | | **t-value (df = 101)** | **CWP** | **Correlation coefficient (R^2^)** |
| --- | --- | --- | --- | --- | --- | --- | --- | --- | --- | --- |
| **Cortical surface area (left)** |  |  |  |  |  |  |  |  |  |  |
| Lateral prefrontal cortex (lPFC) Medial prefrontal cortex (mPFC) Orbitofrontal cortex (OFC) Anterior cingulate cortex (ACC) | left frontal | yellow | 4759 | 6554 | -23.3 | 61.3 | 13.1 | 3.662 | 0.0002 | -0.34 (0.12) |
| Postcentral gyrus (PoCG) | left parietal | blue | 2686 | 5799 | -61.6 | -26.8 | 28.2 | 2.946 | 0.002 | -0.28 (0.08) |
| Inferior temporal gyrus (ITG) | left temporal | red | 2883 | 4728 | -20.6 | -13.0 | -30.1 | 3.173 | 0.001 | -0.3 (0.09) |
| **Cortical surface area (right)** |  |  |  |  |  |  |  |  |  |  |
| Postcentral gyrus (PoCG) Precentral gyrus (PrCG) Superior parietal lobule (SPL) | right frontoparietal | blue | 4002 | 8743 | 42.5 | -30.4 | 57.6 | 3.662 | 0.0002 | -0.34 (0.12) |
| Inferior temporal gyrus (ITG) | right temporal | red | 2387 | 3427 | 56.8 | -33.6 | -16.6 | 2.405 | 0.009 | -0.23 (0.05) |
| Precuneus (PCUN) | right precuneus | green | 2638 | 3864 | 8.7 | -72.4 | 51 | 2.625 | 0.005 | -0.25 (0.06) |
| MNI coordinates = coordinates of the maximum value found in the cluster within the MNI space; CWP = Clusterwise corrected p-value. [Only clusters exceeding a clusterwise corrected probability CWP of p < 0.05 are described]. | | | | | | | | | | |

**REFERENCES**

Balzer, C., Berger, J.-M., Caprez, G., Gonser, A., Gutbrod, K., and Keller, M. (2011). *Materialien und Normwerte für die neuropsychologische Diagnostik MNND. Testhandbuch.* Rheinfelden: Verlag Normdaten.

Behrens, T.E., Woolrich, M.W., Jenkinson, M., Johansen-Berg, H., Nunes, R.G., Clare, S., Matthews, P.M., Brady, J.M., and Smith, S.M. (2003). Characterization and propagation of uncertainty in diffusion-weighted MR imaging. *Magn Reson Med* 50**,** 1077-1088. doi: 10.1002/mrm.10609.

Benton, A.L., Hamsher, K.d.S., and Sivan, A.B. (1994). *Multilingual aphasia examination (third edition).* San Antonio, TX: Psychological Corporation.

Dale, A.M., Fischl, B., and Sereno, M.I. (1999). Cortical surface-based analysis. I. Segmentation and surface reconstruction. *Neuroimage* 9**,** 179-194. doi: 10.1006/nimg.1998.0395.

Desikan, R.S., Segonne, F., Fischl, B., Quinn, B.T., Dickerson, B.C., Blacker, D., Buckner, R.L., Dale, A.M., Maguire, R.P., Hyman, B.T., Albert, M.S., and Killiany, R.J. (2006). An automated labeling system for subdividing the human cerebral cortex on MRI scans into gyral based regions of interest. *Neuroimage* 31**,** 968-980. doi: 10.1016/j.neuroimage.2006.01.021.

Destrieux, C., Fischl, B., Dale, A., and Halgren, E. (2010). Automatic parcellation of human cortical gyri and sulci using standard anatomical nomenclature. *Neuroimage* 53**,** 1-15. doi: 10.1016/j.neuroimage.2010.06.010.

Fischl, B., and Dale, A.M. (2000). Measuring the thickness of the human cerebral cortex from magnetic resonance images. *Proc Natl Acad Sci U S A* 97**,** 11050-11055. doi: 10.1073/pnas.200033797.

Fischl, B., Salat, D.H., van der Kouwe, A.J., Makris, N., Segonne, F., Quinn, B.T., and Dale, A.M. (2004a). Sequence-independent segmentation of magnetic resonance images. *Neuroimage* 23 Suppl 1**,** S69-84. doi: 10.1016/j.neuroimage.2004.07.016.

Fischl, B., Sereno, M.I., and Dale, A.M. (1999a). Cortical surface-based analysis. II: Inflation, flattening, and a surface-based coordinate system. *Neuroimage* 9**,** 195-207. doi: 10.1006/nimg.1998.0396.

Fischl, B., Sereno, M.I., Tootell, R.B., and Dale, A.M. (1999b). High-resolution intersubject averaging and a coordinate system for the cortical surface. *Hum Brain Mapp* 8**,** 272-284. doi: 10.1002/(SICI)1097-0193(1999)8:4<272::AID-HBM10>3.0.CO;2-4.

Fischl, B., van der Kouwe, A., Destrieux, C., Halgren, E., Segonne, F., Salat, D.H., Busa, E., Seidman, L.J., Goldstein, J., Kennedy, D., Caviness, V., Makris, N., Rosen, B., and Dale, A.M. (2004b). Automatically parcellating the human cerebral cortex. *Cereb Cortex* 14**,** 11-22. doi: 10.1093/cercor/bhg087.

Formann, A.K., Waldherr, K., and Piswanger, K. (2011). *WMT-2. Wiener Matrizen-Test 2. Ein Rasch-skalierter spreachfreier Kurztest zur Erfassung der Intelligenz. Manual.* Göttingen: Beltz Test Gesellschaft.

Kuperberg, G.R., Broome, M.R., McGuire, P.K., David, A.S., Eddy, M., Ozawa, F., Goff, D., West, W.C., Williams, S.C.R., van der Kouwe, A.J.W., Salat, D.H., Dale, A.M., and Fischl, B. (2003). Regionally Localized Thinning of the Cerebral Cortex in Schizophrenia. *Arch Gen Psychiatry* 60**,** 878-888. doi: 10.1001/archpsyc.60.9.878.

Park, J.H., Park, S.W., Kang, S.H., Nam, T.K., Min, B.K., and Hwang, S.N. (2009). Detection of traumatic cerebral microbleeds by susceptibility-weighted image of MRI. *J Korean Neurosurg Soc* 46**,** 365-369. doi: 10.3340/jkns.2009.46.4.365.

Raven, J. (1958). *Standard progressive matrices.* London: Lewis & Co.

Rey, A. (1959). *Manuel du test de copie d'une figure complexe de A. Rey.* Paris: Les Editions du Centre de Psychologie Appliquée.

Rosas, H.D., Liu, A.K., Hersch, S., Glessner, M., Ferrante, R.J., Salat, D.H., van der Kouwe, A., Jenkins, B.G., Dale, A.M., and Fischl, B. (2002). Regional and progressive thinning of the cortical ribbon in Huntington's disease. *Neurology* 58**,** 695-701. doi: 10.​1212/​WNL.​58.​5.​695.

Rosenbaum, S.B., and Lipton, M.L. (2012). Embracing chaos: the scope and importance of clinical and pathological heterogeneity in mTBI. *Brain Imaging Behav* 6**,** 255-282. doi: 10.1007/s11682-012-9162-7.

Salat, D.H., Buckner, R.L., Snyder, A.Z., Greve, D.N., Desikan, R.S.R., Busa, E., Morris, J.C., Dale, A.M., and Fischl, B. (2004). Thinning of the Cerebral Cortex in Aging. *Cereb. Cortex* 14**,** 721-730. doi: 10.1093/cercor/bhh032.

Smith, S.M., Jenkinson, M., Woolrich, M.W., Beckmann, C.F., Behrens, T.E., Johansen-Berg, H., Bannister, P.R., De Luca, M., Drobnjak, I., Flitney, D.E., Niazy, R.K., Saunders, J., Vickers, J., Zhang, Y., De Stefano, N., Brady, J.M., and Matthews, P.M. (2004). Advances in functional and structural MR image analysis and implementation as FSL. *Neuroimage* 23 Suppl 1**,** S208-219. doi: 10.1016/j.neuroimage.2004.07.051.

Strauss, E., Sherman, E.M.S., and Spreen, O. (2006). *A compendium of neuropsychological tests. Administration, norms, and commentary (third edition)* New York: Oxford University Press.

Tzourio-Mazoyer, N., Landeau, B., Papathanassiou, D., Crivello, F., Etard, O., Delcroix, N., Mazoyer, B., and Joliot, M. (2002). Automated anatomical labeling of activations in SPM using a macroscopic anatomical parcellation of the MNI MRI single-subject brain. *Neuroimage* 15**,** 273-289. doi: 10.1006/nimg.2001.0978.

Von Aster, M., Neubauer, A., and Horn, R. (2006). *WIE. Wechsler Intelligenztest für Erwachsene. Deutschsprachige Bearbeitung und Adaptation des WAIS II von David Wechsler.* Frankfurt a. M.: Harcourt Test Services.

Wechsler, D. (1997). *Wechsler Adult Intelligence Scale - Third Edition.* San Antonio, TX: The Psychological Corporation.

Zalesky, A., Fornito, A., and Bullmore, E.T. (2010). Network-based statistic: identifying differences in brain networks. *Neuroimage* 53**,** 1197-1207. doi: 10.1016/j.neuroimage.2010.06.041.

Zimmermann, P., and Fimm, B. (2002). "A test battery for attentional performance," in *Applied Neuropsychology of Attention. Theory, Diagnosis and Rehabilitation,* eds. Leclercq M. & Z. P. (London: Psychology Press), 110-151.
